# Management of Hypercholesterolemia Through Dietary ß-glucans–Insights From a Zebrafish Model

**DOI:** 10.3389/fnut.2021.797452

**Published:** 2022-01-12

**Authors:** Adnan Hussain Gora, Saima Rehman, Viswanath Kiron, Jorge Dias, Jorge M. O. Fernandes, Pål Asgeir Olsvik, Prabhugouda Siriyappagouder, Ioannis Vatsos, Ulrike Schmid-Staiger, Konstantin Frick, Miguel Cardoso

**Affiliations:** ^1^Faculty of Biosciences and Aquaculture, Nord University, Bodø, Norway; ^2^SPAROS Lda., Olhão, Portugal; ^3^Fraunhofer Institute for Interfacial Engineering and Biotechnology IGB, Innovation Field Algae Biotechnology-Development, Stuttgart, Germany; ^4^Institute of Interfacial Process Engineering and Plasma Technology, University of Stuttgart, Stuttgart, Germany; ^5^MadeBiotech and NatureXtracts S.A., Caniçal, Portugal

**Keywords:** β-glucans, cholesterol, lipids, microalgae, RNA-Seq, zebrafish

## Abstract

Consumption of lipid-rich foods can increase the blood cholesterol content. β-glucans have hypocholesterolemic effect. However, subtle changes in their molecular branching can influence bioactivity. Therefore, a comparative investigation of the cholesterol-lowering potential of two β-glucans with different branching patterns and a cholesterol-lowering drug, namely simvastatin was undertaken employing the zebrafish (*Danio rerio*) model of diet-induced hypercholesterolemia. Fish were allocated to 5 dietary treatments; a control group, a high cholesterol group, two β-glucan groups, and a simvastatin group. We investigated plasma total cholesterol, LDL and HDL cholesterol levels, histological changes in the tissues, and explored intestinal transcriptomic changes induced by the experimental diets. Dietary cholesterol likely caused the suppression of endogenous cholesterol biosynthesis, induced dysfunction of endoplasmic reticulum and mitochondria, and altered the histomorphology of the intestine. The two β-glucans and simvastatin significantly abated the rise in plasma cholesterol levels and restored the expression of specific genes to alleviate the endoplasmic reticulum-related effects induced by the dietary cholesterol. Furthermore, the distinct patterns of transcriptomic changes in the intestine elicited by the oat and microalga β-glucans impacted processes such as fatty acid metabolism, protein catabolic processes, and nuclear division. Oat and microalgal β-glucans also altered the pattern of lipid deposition in the liver. Our study provides insights into the effectiveness of different β-glucans to alleviate dysfunctions in lipid metabolism caused by dietary cholesterol.

## Introduction

The drastic shift in the global food system has driven a new trend in consuming calorie-rich and highly processed foods (1). Increased consumption of lipid-rich diets is directly correlated with the risk of cardiovascular diseases (CVDs) (2, 3). Cholesterol is one of the main components in the western diet, but it is not an essential nutrient for vertebrates as this form of lipid can be synthesized *de novo* (4). However, cholesterol obtained from diets is known to have a significant impact on lipoproteins and their levels in the blood (5). The two classes of lipoproteins, namely high density lipoprotein (HDL) and low density lipoprotein (LDL) have distinct properties. LDL is considered as the main proatherogenic lipoprotein. Accumulation and subsequent oxidation of LDL in the arterial wall triggering an inflammatory response is a critical step toward the development of atherosclerosis (6). On the other hand, HDL performs cholesterol efflux from peripheral tissues to the liver, and the lipoprotein is known to possess anti-inflammatory and antioxidative effects as well as LDL oxidation lowering ability (7). The liver and intestine are the two principal organs that regulate the circulating lipoproteins and cholesterol homeostasis in the body, and specific receptors are known to facilitate metabolism linked to bile and dietary cholesterol (8, 9). The dietary cholesterol, along with other lipids and the synthesized apolipoproteins forms chylomicrons (10, 11), which are finally released into the blood. The endogenous pathway of cholesterol metabolism generates LDLs and the amounts of LDL cholesterol present in the circulation is largely determined by the rate of uptake by specific receptors in the liver (12). If the cholesterol content of the hepatocytes is high, LDL receptor activity is decreased (13), causing a reduction in the uptake of LDL by the hepatocytes. This in turn increases the amounts of LDL in circulation. Higher LDL cholesterol content in the blood is directly associated with lifestyle diseases like ischemic heart disease and stroke (3, 14).

Dietary and endogenous factors are known to adversely affect cholesterol uptake and biosynthesis, but some diet components, paradoxically, can stall the progression of lifestyle diseases by regulating the total circulating cholesterol and LDL-cholesterol levels. In fact, encouraging outcomes through dietary and therapeutic interventions have spurred an interest in developing strategies to manage LDL-cholesterol through diet. Targeting LDL-cholesterol reduction through diets is a better approach because medicines are associated with many side effects. Statins are a class of drugs commonly used to manage hypercholesterolemia. These drugs though effective, exhibit myotoxic side effects which include myalgia, myositis and rhabdomyolysis (15, 16). As an alternative, dietary intervention can be adopted to maintain cholesterol homeostasis. The benefits of using natural bioactive compounds are multifaceted if they are employed to arrest hypercholesterolemia (17, 18). β-glucans found in plants, microalgae and fungi have several unexploited properties, and subtle changes in their structural organization can elevate their efficacy. For example, fungal β-glucans, with β-(1, 3) backbone and (1, 6) linkages, have immunostimulatory and anti-tumor properties (19). In contrast, β-glucan from cereals, which have β-(1, 4) linkages, help lower cholesterol and blood glucose (20). The marine microalga *Phaeodactylum tricornutum*, is a rich source of β-glucan (21), and this polysaccharide has a linear chain of β-(1, 3) linkage and branching at the C-6 position (22). Studies have reported that the functionality and biopotency of β-glucans are largely determined by their molecular structure (23). Although branching differences between microalgal β-glucans and oat glucans are well known (24), it is unclear how these structural differences affect bioactivity *in vivo*. It is plausible that β-glucans from different sources are recognized by specific receptors, the affinity of which varies based on the molecular structure of the β-glucans (25). In addition, each β-glucan type may have its distinct gel-forming ability (26), prebiotic property (27) and influence on microbiota to affect the blood cholesterol levels (28). Moreover, we cannot discount the effect of interaction of these polysaccharides with other food components. Consumption of oat β-glucan with food and juices lead to different outcomes in humans. For example, beverages enriched with oat is effective in decreasing the LDL or total cholesterol in humans (29), whereas bread with oat β-glucan can decrease LDL cholesterol, total/HDL cholesterol ratio and LDL/HDL cholesterol ratio (30) indicating the impact of the food matrix on the efficacy of β-glucans.

A comparative investigation of dietary β-glucans from two sources with different molecular structures was undertaken employing a zebrafish model of hypercholesterolemia. Zebrafish is considered a valuable model species to understand abnormalities in lipid metabolism that result in disease progression. Fundamental lipoprotein pathways are conserved in humans and zebrafish (31) and adult zebrafish fed a cholesterol-rich diet are highly susceptible to hypercholesterolemia (32, 33). This model also allowed us to test the therapeutic potential of two dietary components which can eventually be considered as hypercholesterolemia-controlling agents in humans (34). We targeted the intestine tissue because of its importance in dietary lipid uptake and regulation of cholesterol metabolism (35).

We hypothesized that dietary β-glucans with different molecular structures may influence the circulating cholesterol levels and their associated responses in the intestine. RNA sequencing was employed to understand the underlying impact of dietary cholesterol, β-glucans and simvastatin on the intestinal cholesterol metabolism of zebrafish at a molecular level.

## Materials and Methods

### Experimental Fish

Three hundred male (14-month-old) zebrafish, *Danio rerio*, were used for the experiment. To obtain this experimental fish stock, the adult fish were bred in-house in the zebrafish facility of Nord University, Norway, following standard protocols (36). The eggs were maintained in E3 medium and incubated at 28 °C in an incubator until hatching i.e., at around 50 h post-fertilization. From 4 to 14 days post-fertilization, the larvae were fed the commercial micro diet Zebrafeed® (SPAROS Lda, Olhão, Portugal) of <100 μm particle size and *Artemia nauplii, ad libitum*. From 15 days post-fertilization (advanced larval stage) onwards, they were fed only micro diets of 100-200 μm particle size (Zebrafeed®). On month 14, the fish were randomly distributed into 30 tanks (6 tanks per treatment group) of a freshwater flow-through system (Zebtec Toxicological Rack, Tecniplast, Varese, Italy) with 3.5 L tank capacity. The stocking density was ten fish per tank. The fish were acclimatized in the flow-through system for 2 weeks and were fed control diet during this period. The water temperature in the tanks was 28 ± 0.5°C, and the water flow rate was 2.5 L/h. The dissolved oxygen in the tanks ranged between 7 and 8 ppm (oxygen saturation > 85%). A 14L:10D photoperiod was maintained throughout the experimental period.

### β-Glucans, Diets and Feeding Experiment

The alga β-glucans employed in the present study originated from the microalga *Phaeodactylum tricornutum*, strain SAG 1090-1b (culture collection from the University of Goettingen) also designated as CCAP strain 1052/1B or UTEX 640. *P. tricornutum* (SAG 1090 1b) grown under nitrogen-depleted conditions in flat panel airlift reactors was harvested, concentrated via centrifugation to 250–270 g L^−1^ (Clara 20, Alfa Laval) and frozen at −20°C. For further processing, the biomass was thawed and diluted to 100 g L^−1^ with deionized water. After disrupting the algal cells with a ball mill (PML-2, Bühler), the biomass was centrifuged and the supernatant, containing the enriched fraction of β-glucan, was freeze-dried (Cat. Number: J326XP-IM-4, Avanti J-26 XP, Beckman Coulter). Besides β-glucan, the alga product contained a low level of protein (3.7%) and fat (<0.5%), and the remaining fraction could be presumed as other soluble carbohydrates. The oat β-glucans used in the study was a commercial product PromOat® (Lantmännen Oats AB, Sweden). According to the manufacturer, the oat β-glucan product also contains high levels of other carbohydrates including starch and low amounts of protein (4%) and fat (0.5%). SPAROS Lda. prepared the five experimental diets (Supplementary Table 1): 4 high-cholesterol diets and 1 control diet. The high cholesterol diet (HC) had 5.1% inclusion of purified cholesterol. We selected this level based on previous studies on zebrafish (32, 33). A standard low cholesterol diet without supplementation of purified cholesterol served as the control diet (CT). The fatty acids in the two basal diets, CT and HC (Supplementary Table 2) were profiled by Eurofins Food Testing Lisboa (Alcochete, Portugal).

The algal (AG) and oat glucan (OG) diets had 2.5% (with 30% purity, final content 0.8%) each of alga-derived and oat-derived β-glucans, respectively incorporated into the HC diet. The SS diet contained 50 mg kg^−1^ of simvastatin (Cat. Number: S6196, SigmaAldrich, St. Louis, MO, USA) in the HC diet; the inclusion level was based on a previous study (37). Thus AG, OG and SS diets had all the ingredients in the HC diet in addition to the respective test compound. The daily feeding rate was 4% of total biomass per day. The fish were fed thrice a day during the 12-week feeding trial; at 08:00, 13:00, and 18:00.

### Sampling

At the end of the experimental period, fish were sacrificed by immersing in 200 mg L^−1^ of tricaine methane sulfate (Cat. Number: E10521, SigmaAldrich), which was buffered with 200 mg L^−1^ of sodium bicarbonate (Cat. Number: S5761, SigmaAldrich). Blood drawn by tail ablation method (38) was collected in a heparinized tube, and was centrifuged at 5,000 *g* for 10 min at 4 °C to collect the plasma (*n* = 5 per group; 5 fish from each tank pooled). The middle intestine (*n* = 5 to 6 per group) was carefully dissected and snap-frozen in liquid nitrogen followed by storage at −80°C until use.

### Plasma Total Cholesterol, LDL Cholesterol and HDL Cholesterol Estimation

The total, LDL, and HDL cholesterol levels in the plasma were estimated using the HDL and LDL/VLDL Cholesterol Assay Kit (Cat. Number: ab65390, Abcam, Cambridge, UK). Plasma from five fish per tank was pooled and considered as one replicate sample. Two microliters of this pooled plasma were used for quantifying the total, LDL- and HDL-cholesterol content, following the manufacturer's instructions.

### Transcriptomic Analysis of Intestine

To extract total RNA, the frozen intestine samples were briefly homogenized in QIAzol lysis reagent (Cat. Number: 79306, Qiagen, Hilden, Germany) at 6,500 rpm for 2 × 20 s in a Precellys 24 homogenizer (Cat. Number: P000669-PR240-A, Bertin Instruments, Montigny-le-Bretonneux, France). RNA was extracted from the tissue homogenate using Direct-zol™ RNA MiniPrep (Cat. Number: R2052, Zymoresearch, CA, USA) following the manufacturer's instructions. The RNA concentration and integrity were determined using Qubit 4 Fluorometer (Cat. Number: Q33238, Thermo Fisher Scientific, Waltham MA, USA) and Tape Station 2200 (Cat. Number: G2964AA, Agilent Technologies, Santa Clara, CA, USA). Only RNA samples exhibiting RIN value >7 were used to construct the RNA-Seq libraries. Libraries were prepared as described in our previous publication (39) using the NEBNext Ultra™ RNA Library Prep Kit (Cat. Number: E7760S, NE Biolabs, Ipswich, MA, USA) with the poly (A) mRNA magnetic isolation module (Cat. Number: E7490S, NE Biolabs). Briefly, one μg of total RNA was used for library preparation and after Poly(A) enrichment, mRNA was fragmented to 100–200 nt length. Next, the first and second strands of cDNA was synthesized, and then the cDNA was purified, end-repaired and used for adaptor ligation followed by barcoding using NEBNext Multiplex Oligos (Cat. Numbers: E7600S and E7780S, NE Biolabs). PCR enrichment was done for nine cycles, and the amplified libraries were purified using AMPure XP beads (Cat. Number: A63881, Beckman Coulter, Inc., Brea, USA). The barcoded libraries were then pooled and loaded at 1.4pM on the Illumina NextSeq 500 sequencer (Cat. Number: SY-415-1001, Illumina, San Diego, CA, USA) using the NextSeq 500/550 High Output Kit (Cat. Number: FC-404-2005, Illumina) for 75 bp single-end sequencing at the genomics platform of Nord University (Bodø, Norway). The average mapping percentage for the whole data set was 91.4% (Supplementary Table 3).

### Bioinformatic Analysis

The quality of the reads was assessed using the *fastQC* command. Adapter sequences and low quality reads (Phred quality score, Q<30) were trimmed from the raw reads using the fastp software (40). The reads were then aligned to the reference zebrafish genome downloaded from NCBI (release 100) using HISAT2, version 2.2.1, which uses an indexed reference genome for alignment (41). The reads were annotated using *featureCounts* to obtain the read counts that belong to each gene (42). Differential expression analyses of the genes across the treatment groups was performed using the R package *DESeq2* (version 1.30.0). Transcripts with an absolute Log_2_ fold change of 1 and an adjusted *p*-value (q-value) of < 0.05 (Benjamini-Hochberg multiple test correction method) were considered significantly differentially expressed and used for gene ontology (GO) and KEGG pathway analysis. The gene ontology enrichment was performed with Database for Annotation, Visualization and Integrated Discovery (*DAVID*) version 6.8 and the *clusterProfiler* package (version 3.18.0) in R. The packages *ggplot2* (version 3.3.3), *pheatmap* (version 1.0.12) and *GOplot* (version 1.0.2) were employed to visualize the data. Gene ontology networks were generated using Cytoscape (43) (version 3.8.2). In addition, long lists of gene ontology terms were summarized into non-redundant terms using the REViGO online tool (44).

### Histological Analysis of the Liver and the Intestine

The liver and the mid-intestine samples (*n* = 8 per group) were fixed in 3.7 % (v/v) phosphate-buffered formaldehyde solution (pH 7.2) at 4 °C for 24 h. Standard histological procedures were followed for dehydration, processing, and paraffin embedding as described by Bancroft and Gamble (45). The paraffin blocks thus prepared were sectioned using a microtome (Microm HM355S, MICROM International GmbH, Walldorf, Germany). Four micrometer thick longitudinal sections were cut and mounted on SuperFrost® slides (Menzel, Braunschweig, Germany). A robot slide stainer Microm HMS 760X (MICROM International GmbH) was used to stain the intestine sections with Alcian Blue (Cat. Number: A3157, SigmaAldrich) and Periodic Acid Schiff's Reagent (Cat. Numbers: 375810 and 1.09033, SigmaAldrich)(AB-PAS, pH 1) and the liver sections with hematoxylin (Cat. Number: H9627, SigmaAldrich) and eosin (Cat. Number: 861006, SigmaAldrich). Light microscopy photomicrographs were taken with the Leica DM3000 LED microscope (Leica Camera AG, Wetzlar, Germany) fitted with Leica MC 190HD camera (Leica Camera AG). The software ImageJ (46) was used for quantifying the histological indices. The histological indices that were evaluated were: length of the villi, width of lamina propria, submucosa thickness and tunica muscularis thickness, as described in our previous publication (47) (Supplementary Figure 1). Liver vacuolation was assessed by evaluating two parameters-average lipid vacuole area and average lipid vacuole number in a selected area of the liver (Supplementary Figure 2). Shapiro-Wilk test and Bartlett's test were employed to confirm the data normality and homoscedasticity, respectively. Parametric *t*-test and one-way ANOVA were performed where the normality assumptions were met. In the case of non-parametric data, statistical differences were identified using the Wilcoxon-Mann-Whitney test and Kruskal-Wallis test. Tukey's test (parametric data) and Dunn's test (non-parametric data) were employed to understand the differences between groups of interest.

### qPCR Verification of the RNA Seq Results

Differential expression of selected genes from the transcriptome data was verified by qPCR analysis. The same samples used for RNA-Seq were employed for qPCR-based verification, and reactions were run with a sample size of 5 per group. One μg of total RNA from each sample was reverse transcribed using the QuantiTect reverse transcription kit (Cat. Number: 205311, Qiagen), according to the manufacturer's instructions. The cDNA was further diluted ten times with nuclease-free water and used as a PCR template. The PCR reactions were conducted using the SYBR green (Cat. Number: 04707516001, Roche Holding AG, Basel, Switzerland) in LightCycler® 96 Real-Time PCR System (Cat. Number: 05815916001, Roche Holding). Relative expression of selected genes was determined based on the geometric mean of reference genes (*actb1, eef1a* and *rpl13*α), for which we employed the primers that are reported previously (48). We designed the primers for the selected genes using the Primer-BLAST tool in NCBI. The primers were then checked for secondary structures such as hairpin, repeats, self and cross dimer by NetPrimer (Premier Biosoft, Palo Alto, USA). The primers for the target genes are listed in Supplementary Table 4. The data were checked for normality (Shapiro-Wilk test) and homoscedasticity (Bartlett's test), based on which, the statistical difference was determined by Student *t*-test or Welch two-sample *t*-test.

## Results

In the present study, we fed adult zebrafish with a control low cholesterol diet (CT) or high cholesterol diets (HC, AG, OG, SS) that contained 5.1% cholesterol.

### Purified Dietary Cholesterol Altered the Plasma Cholesterol Profile

**Figure 1:**
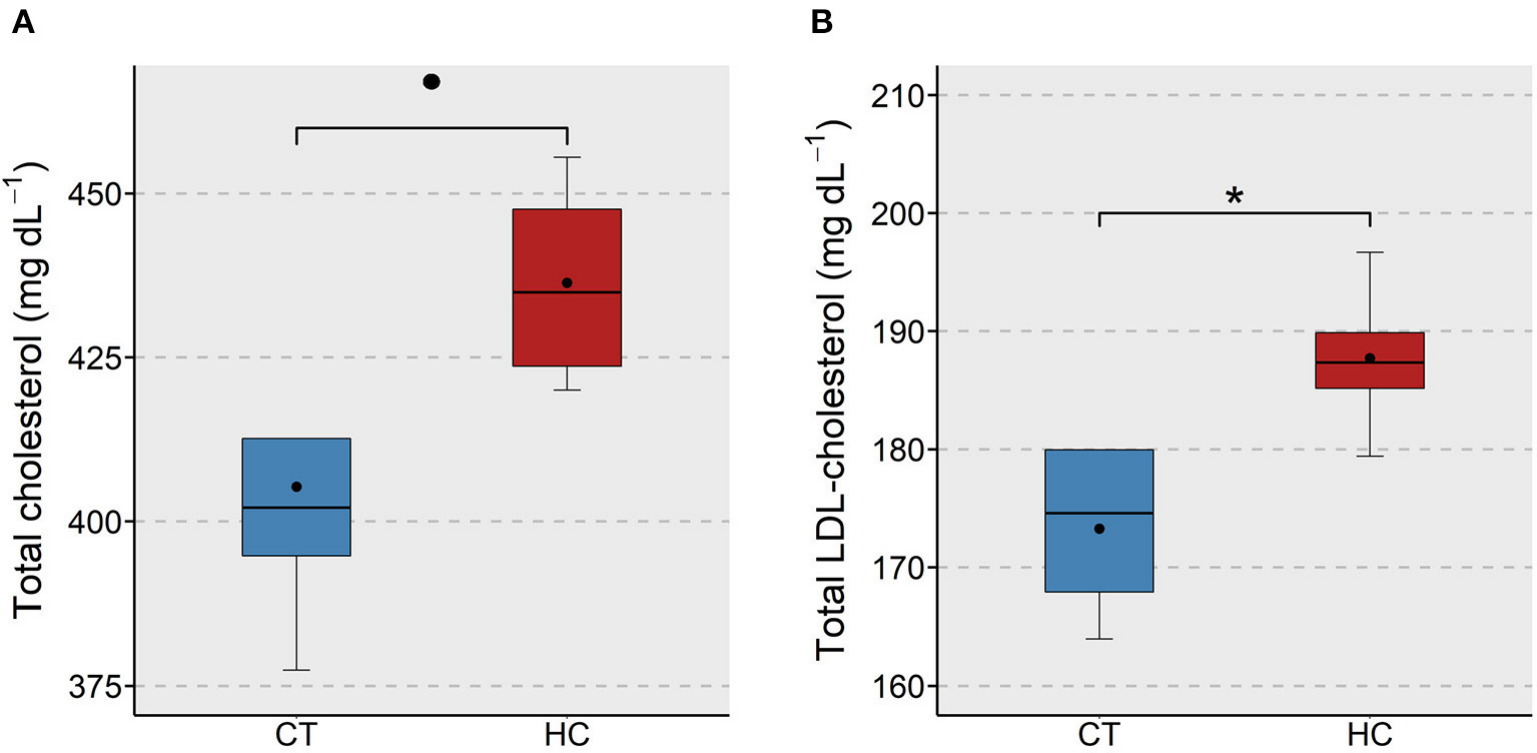
Total and LDL-cholesterol levels in the plasma of zebrafish fed a low or a high cholesterol diet. Boxplots show the total cholesterol **(A)** and LDL-cholesterol **(B)** levels (mg dL^−1^) in the plasma of zebrafish fed either a control (CT) diet or a high cholesterol (HC) diet for a period of 12 weeks. Black dots inside each box indicate the mean values of the corresponding groups. * indicates *p* < 0.05 and • indicates *p* < 0.1.

We examined the effect of purified dietary cholesterol on different cholesterol species that are present in the plasma of adult zebrafish. Total, LDL- and HDL-cholesterol in fish fed the HC diet was compared with those of the CT group. We found an apparent (*p* < 0.1) increase in the total cholesterol content and a significant (*p* < 0.05) increase in the LDL-cholesterol content in the plasma of the HC group (Figures 1A,B). On the other hand, the HDL-cholesterol levels of the two study groups were not significantly different (Supplementary Figure 3A).

### β-Glucans and Simvastatin Reduced the Plasma Cholesterol Levels

**Figure 2:**
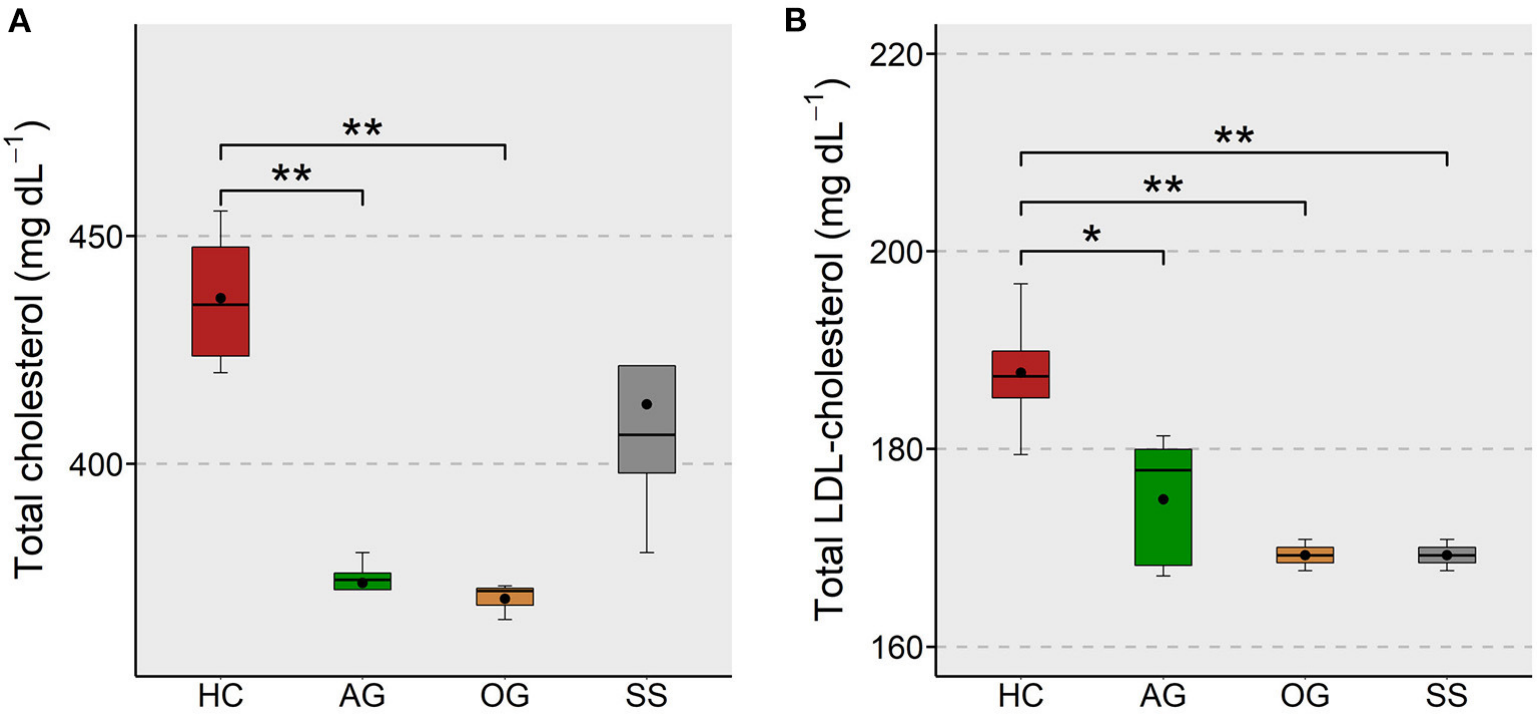
Total and LDL-cholesterol levels in the plasma of zebrafish fed glucans or simvastatin. Boxplots show total cholesterol **(A)** and LDL-cholesterol **(B)** levels (mg dL^−1^) in the plasma of zebrafish fed high cholesterol (HC) diet or HC diet supplemented with either algal glucan (AG) or oat glucan (OG) or simvastatin (SS), for a period of 12 weeks. Black dots inside each box indicate the mean values of the corresponding groups. ^**^indicates *p* < 0.01 and * indicates *p* < 0.05.

The effect of supplementation of different forms of β-glucans and simvastatin on the plasma cholesterol level in zebrafish was assessed to evaluate their effectiveness in keeping the proatherogenic lipoprotein levels under control. We found a significant reduction of total cholesterol in the AG and OG groups compared to the HC group (Figure 2A). The LDL-cholesterol in the AG, OG and SS were lower compared to the HC group (Figure 2B). Again, we did not observe significant differences in the plasma HDL-cholesterol of the treatment groups (Supplementary Figure 3B).

### Dietary Cholesterol Affected Steroid and Bile Biosynthesis and Endoplasmic Reticulum-Linked Genes in the Intestine

**Figure 3:**
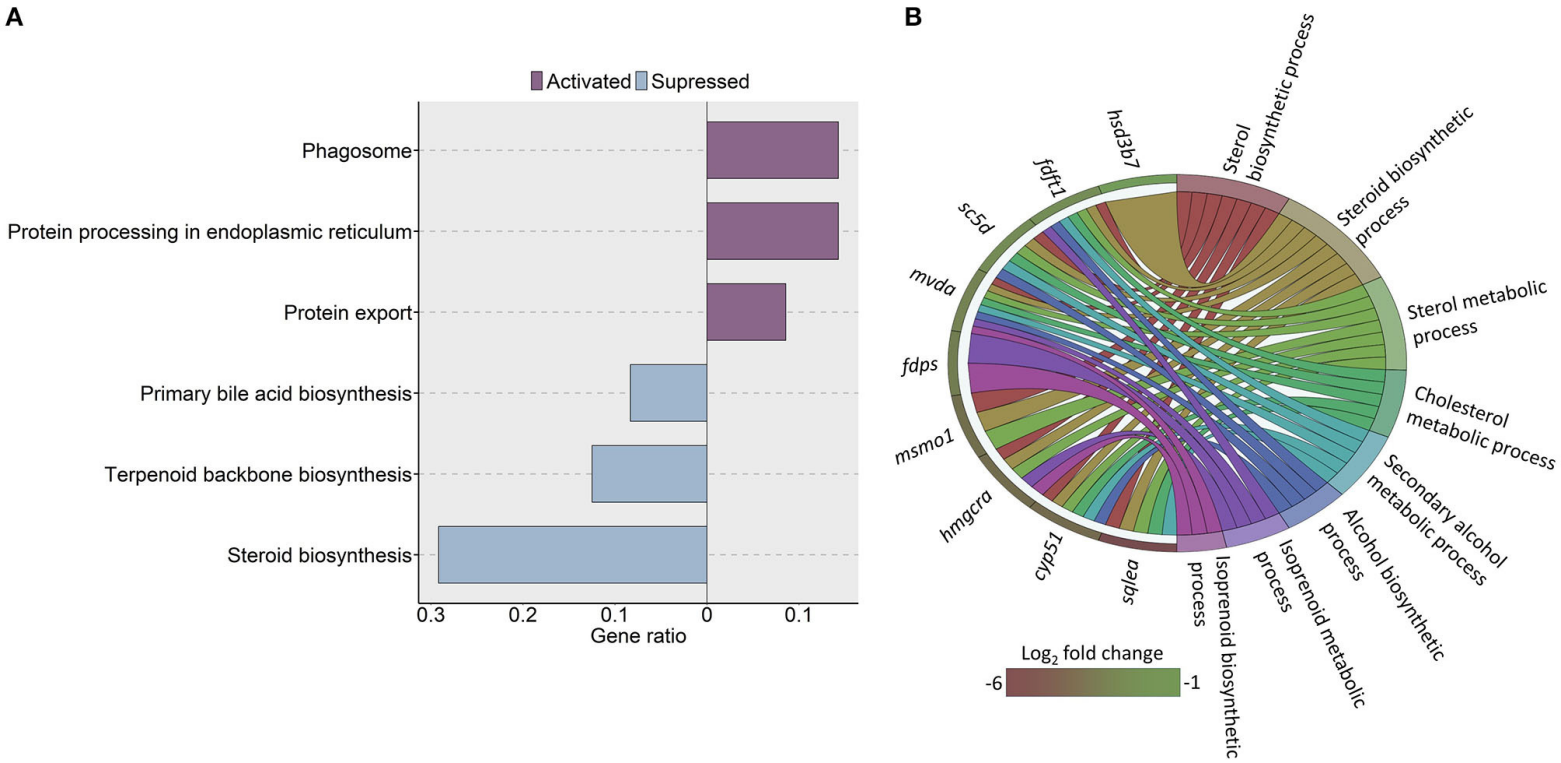
KEGG pathways and GO terms that were enriched in zebrafish fed a high cholesterol diet. **(A)** Significantly altered KEGG pathways in the high cholesterol diet (HC) group, based on the altered genes in the HC group compared to the low cholesterol (CT) diet group. Each treatment group consisted of at least five biological replicates. **(B)** Chord diagram showing the link between the enriched GO terms and the associated genes that were detected as downregulated in the HC vs. CT group comparison. Genes that were considered for the enrichment analyses are shown on the left half of the circle. The gradient color bar intensity varies with the Log_2_ fold change.

To assess whether the cholesterol content in the HC group impacted the cholesterol metabolism in the intestine, we compared the transcriptomes of the HC group and the CT group. The analysis revealed 71 downregulated and 109 upregulated genes (|Log_2_ fold-change| ≥ 1, q-value < 0.05) in the HC group compared to the CT group (Supplementary Table 5). The KEGG pathway enrichment analysis of the downregulated genes revealed significant suppression of steroid biosynthesis, terpenoid backbone biosynthesis and primary bile acid biosynthesis pathways. We found significant enrichment of pathways such as protein export, phagosome, and protein processing in the endoplasmic reticulum (ER) by the upregulated genes (Figure 3A).

**Figure 4:**
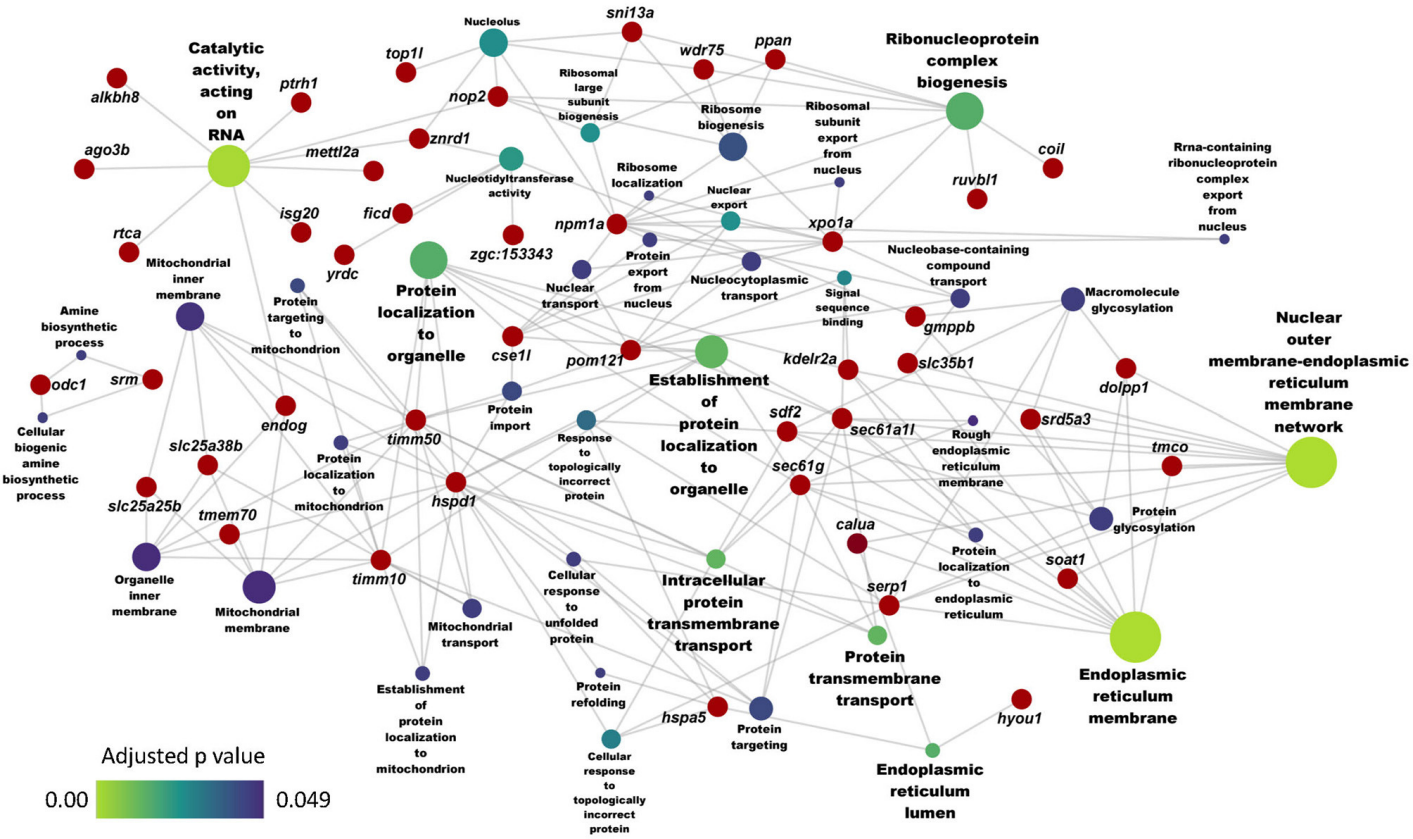
Network showing the link between enriched GO terms and the associated genes that were upregulated in zebrafish fed the high cholesterol (HC) diet compared to the control (CT) diet. Genes that were considered for the enrichment are written near the red circles. The gradient color bar intensity varies with the adjusted *p* value (Benjamini-Hochberg method) for each GO term. Node size of each GO term increases with the number of mapped genes.

Furthermore, the GO analysis revealed significant enrichment of several associated biochemical processes like cholesterol and sterol metabolic processes, sterol and steroid biosynthetic processes because of the downregulated genes in the HC group (Figure 3B). On the other hand, the GO enrichment analysis with the upregulated genes led to the enrichment of ER membrane, ER lumen and nuclear outer membrane reticulum membrane network (Figure 4).

### Algal Glucan Regulated the Expression of Genes Involved in the Intestinal Lipid Metabolism and Vacuole Formation

**Figure 5:**
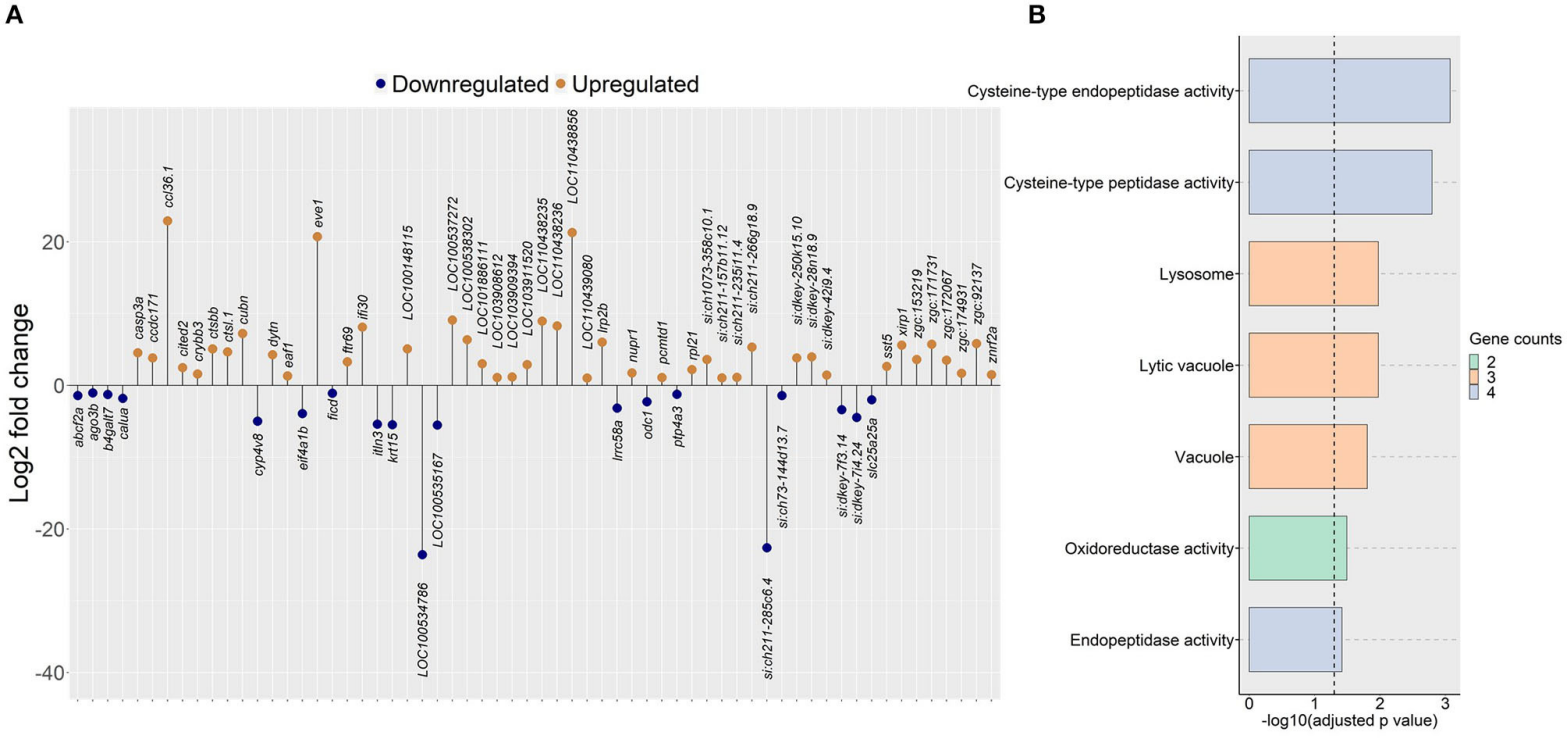
Genes and GO terms that were regulated in zebrafish fed the algal glucan diet. **(A)** Differentially expressed genes obtained by comparing the algal glucan group (AG) with the high cholesterol (HC) group (|Log2 fold-change| ≥ 1, *q*-value < 0.05). **(B)** Bar plot showing enriched GO terms in the AG group, based on genes that were upregulated in the AG group compared to the HC group. Bar colors represent the gene count. For both analyses, each treatment group consisted of at least five biological replicates.

Since the plasma cholesterol levels were affected by algal glucan supplementation in the zebrafish diet, we hypothesized that the algal glucans could alter the cholesterol metabolism in the intestine. To assess this, we compared the intestinal transcriptome of the AG group with that of the HC group. This analysis revealed 19 downregulated and 43 upregulated genes (|Log_2_ fold-change| ≥ 1, q-value < 0.05, Figure 5A, Supplementary Table 6) in the AG group. Several important genes that regulate the lipid metabolism - *cytochrome P450, family 4, subfamily V, polypeptide 8* (*cyp4v8*), *ATP-binding cassette, sub-family F (GCN20), member 2a* (*abcf2a*), vacuole formation-*cathepsin L.1* (*ctsl.1*), *cathepsin Bb* (*ctsbb*)*, IFI30 lysosomal thiol reductase* (*ifi30*) and ER stress-*calumenin a* (*calua*) were differentially regulated by the algal β-glucan. GO analysis based on the upregulated genes revealed significant enrichment of vacuole, lytic vacuole and lysosome (Figure 5B). Our analysis did not detect any significant enrichment of KEGG pathways based on the differentially expressed genes from the AG vs. HC group comparison.

### Oat Glucan Altered Genes Associated With Lipid Metabolism and Endoplasmic Reticulum in the Intestine

**Figure 6:**
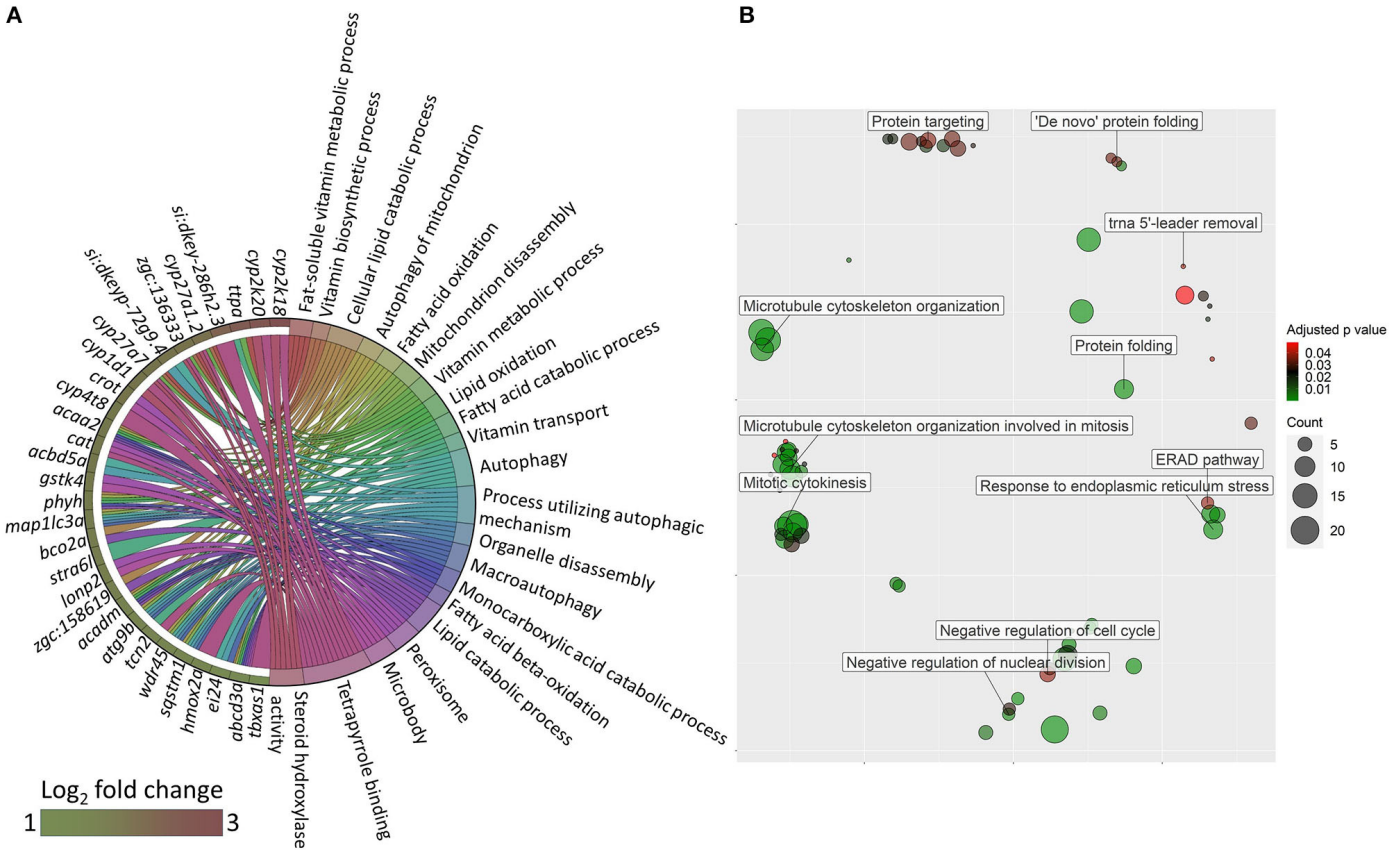
GO terms that were altered in zebrafish fed the oat glucan diet. **(A)** Chord diagram showing the link between enriched GO terms and the associated upregulated genes in the oat glucan (OG) group compared to the high cholesterol diet (HC) group. Genes that were considered for the enrichment are shown on the left half of the circle. The gradient color bar intensity varies with the Log_2_ fold change. **(B)** Enriched GO terms in the OG group. Only the non-redundant GO terms are marked in the bubble plot, and the analysis was performed using the genes that were downregulated in the OG group compared to the HC group. The color of the circles ranging from green to red indicates the order of adjusted *p* value (0.01–0.049). Circle sizes are proportional to the gene count.

**Figure 7:**
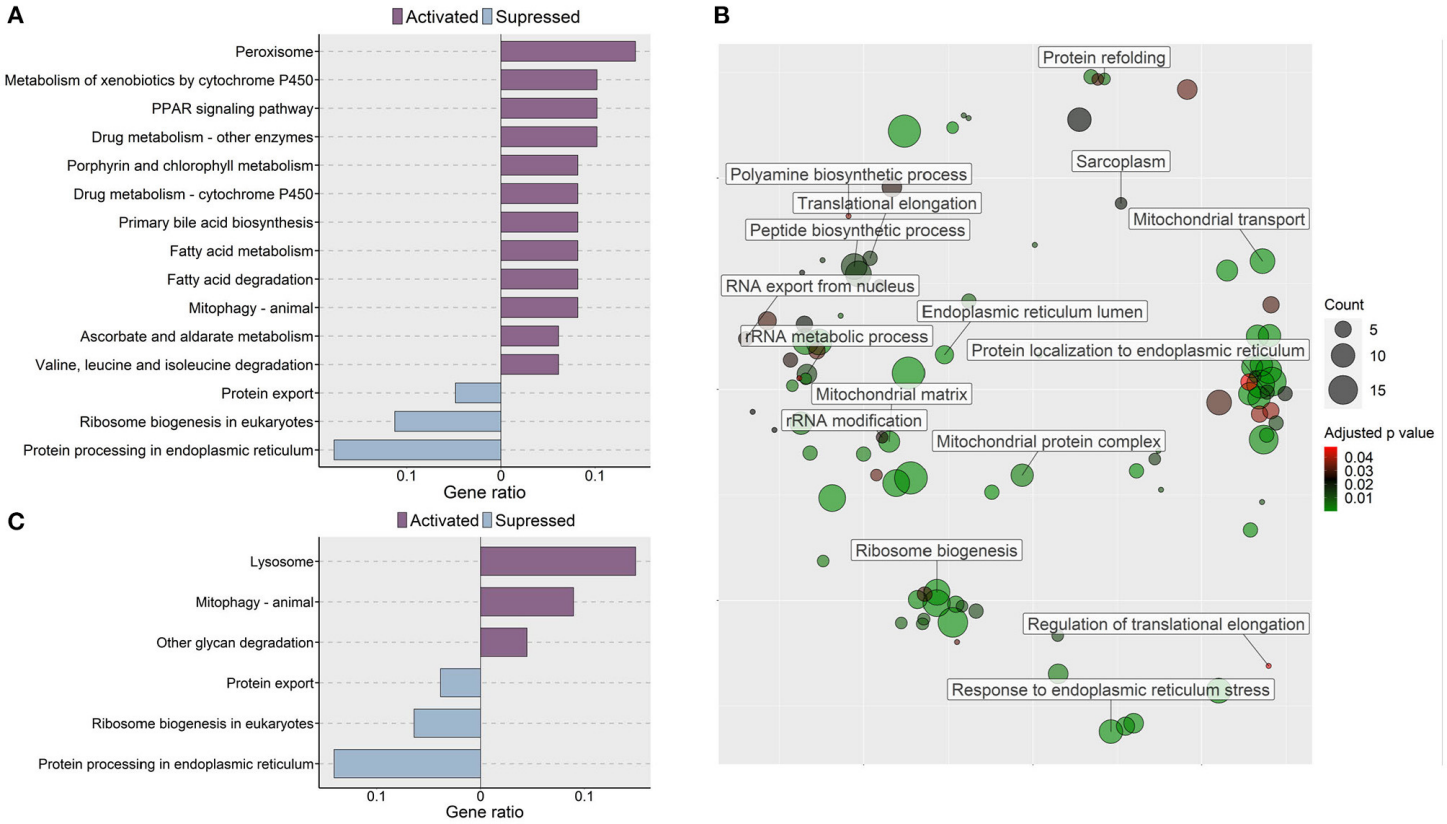
Molecular effects of the oat glucan and simvastatin supplemented diets. **(A)** KEGG pathways that were altered in zebrafish fed the oat glucan (OG) diet. The enrichment analyses were performed by considering the differentially expressed genes in the OG fed zebrafish compared to the fish fed the high cholesterol (HC) diet. **(B)** Enriched GO terms in simvastatin fed (SS) group. Only the non-redundant GO terms are marked in the bubble plot, and the analysis was performed using the genes that were downregulated in the SS group compared to the HC group. The color of the circles ranging from green to red indicates the order of adjusted *p* value. Circle sizes are proportional to the gene number. **(C)** KEGG pathways that were altered in zebrafish fed simvastatin. The enrichment analyses were performed by considering the differentially expressed genes in the SS group compared to the HC group. Each treatment group consisted of at least five biological replicates.

Transcriptomic analysis of the oat glucan group revealed 177 upregulated and 223 downregulated genes (|Log_2_ fold-change| ≥ 1, *q*-value < 0.05, Supplementary Table 7). The gene ontology analysis of the upregulated genes revealed processes like autophagy, steroid hydroxylase activity, lipid catabolic process and lipid oxidation (Figure 6A). The GO analysis of the downregulated genes revealed the suppression of several processes like response to ER stress, ER associated protein degradation (ERAD) pathway and *de novo* protein folding (Figure 6B). KEGG pathway enrichment analysis using the differentially expressed genes revealed the activation (based on the upregulated genes) of PPAR signaling pathway, fatty acid degradation, mitophagy-animal, primary bile acid biosynthesis and the suppression (based on the downregulated genes) of protein export, ribosome biogenesis in eukaryotes, and protein processing in ER (Figure 7A).

### Simvastatin Impacted Genes Connected to Protein Synthesis and Endoplasmic Reticulum in the Intestine

Transcriptomic analysis of the simvastatin fed (SS) group vs. high cholesterol-fed (HC) group revealed 242 upregulated and 224 downregulated genes (|Log_2_ fold-change| ≥ 1, *q*-value < 0.05, Supplementary Table 8). Gene ontology analysis of the downregulated genes revealed the enrichment of several terms like translational elongation, rRNA modification, rRNA metabolic process and peptide biosynthetic process (Figure 7B). KEGG pathway enrichment analysis of the upregulated genes in the SS group revealed activation of lysosome, mitophagy-animal, and other glycan degradation pathways. In contrast, protein export, ribosome biogenesis in eukaryotes and protein processing in ER pathways were likely suppressed by simvastatin feeding (Figure 7C).

### The Intestinal Transcriptome Responds Differentially to Diet-Induced Hypercholesterolemia and Cholesterol-Lowering Agents

**Figure 8:**
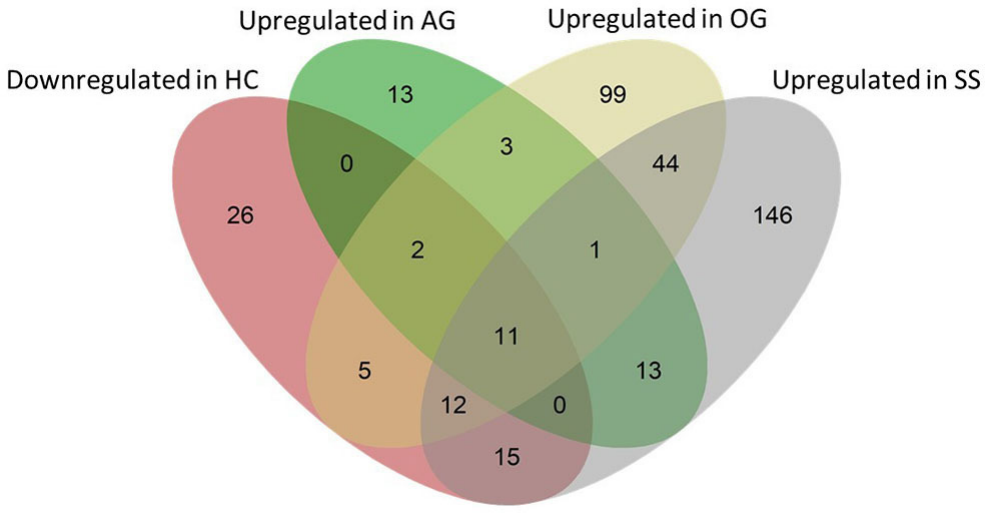
Venn diagram showing the total number of genes that were affected by the experimental diets. The pink ellipse includes the genes that were downregulated in the HC group compared to the CT group. Green, yellow and gray ellipses include genes that were upregulated (compared to the HC group) in the AG, OG and SS groups, respectively. Thirteen (2 + 11), 30 (2 + 5 + 11 + 12) and 38 genes (12 + 15 + 11) that were downregulated in the HC group were upregulated in the AG, OG and SS groups, respectively.

We examined the genes that were downregulated by dietary cholesterol (HC vs. CT) and upregulated by dietary algal glucan (AG vs. HC), oat glucan (OG vs. HC) and simvastatin (SS vs. HC). Algal glucan, oat glucan or simvastatin were able to restore the expression of the genes that were downregulated by cholesterol feeding (Figure 8, Supplementary Figure 4). We found 13 genes downregulated in the intestine when the fish were fed high cholesterol diet, but their expression was restored by algal glucan feeding. Similarly, we found that the expression of 30 genes was restored by oat glucan feeding. Likewise, simvastatin feeding also helped in bringing back the expression of 38 genes. To understand the differential effects of the oat and algal β-glucans, we explored the disparate alteration of gene expression. Among the 124 highly (|Log_2_ fold-change| ≥ 2.5, *q*-value < 0.05) differentially expressed genes obtained from the AG vs. HC and OG vs. HC comparisons, 9 genes were shared and 105 were detected as unique (30 genes in AG vs. HC and 85 genes in OG vs. HC) (Supplementary Figure 5A). Among these 105 uniquely expressed genes, the expression of 95 genes differed in the algal and oat β-glucan fed groups (Supplementary Figure 5B). Twenty-five genes exhibited higher normalized counts in the OG group compared to the AG group. On the other hand, 70 genes exhibited higher normalized counts in the AG group compared to the OG group. Furthermore, the GO analysis of these 95 genes (Supplementary Figure 6) revealed the processes like nuclear division, cellular protein catabolic process, cellular macromolecular catabolic process that were predominantly enriched in group fed algal glucans. On the other hand, GO terms like fatty acid metabolic process, long-chain fatty acid metabolic process and oxoacid metabolic process were predominantly enriched based on the upregulated genes in the OG group compared to the AG group.

### Diets Altered the Vacuolization in the Liver and Micromorphology of the Intestine

**Figure 9:**
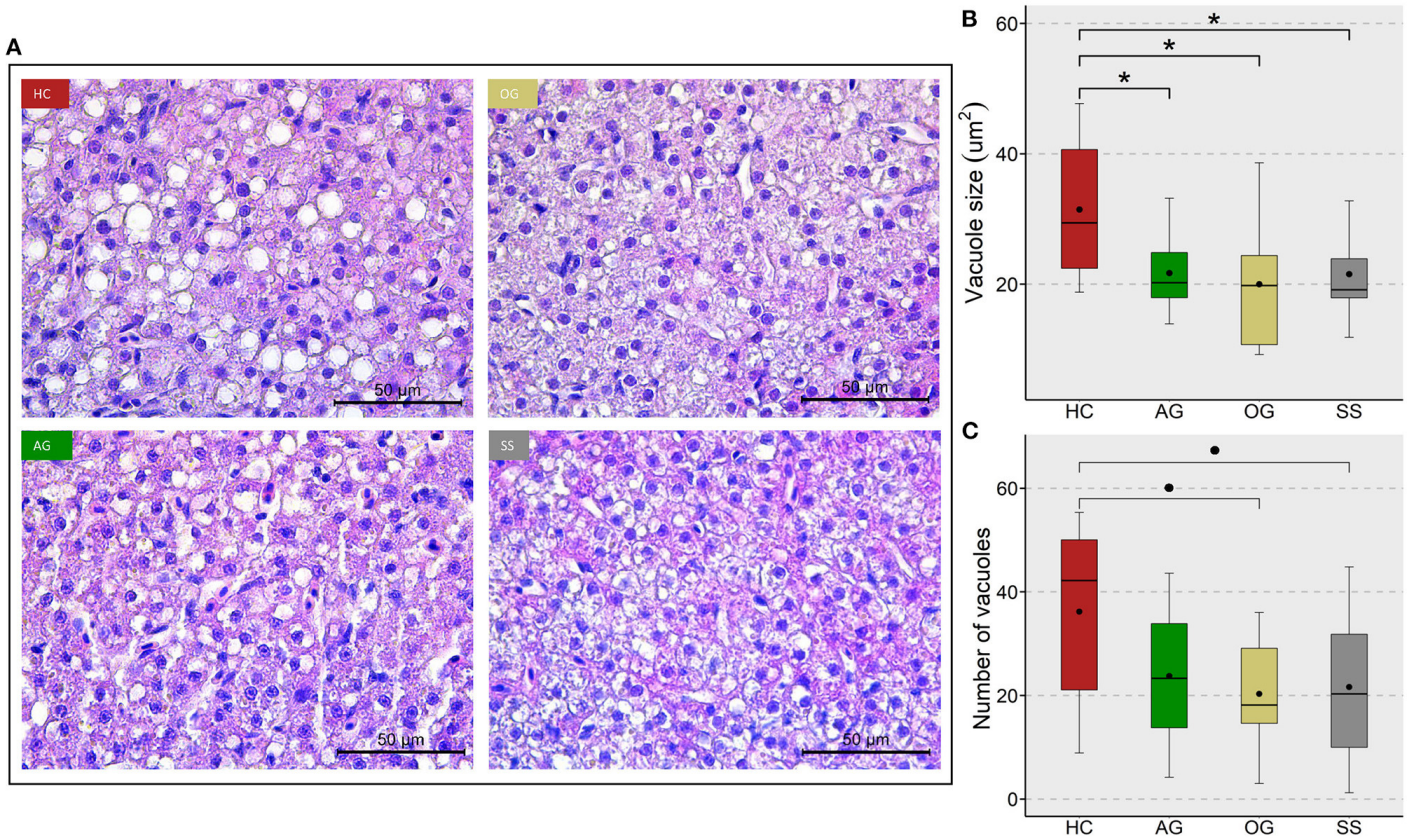
Lipid accumulation in the liver of zebrafish fed four experimental diets. **(A)** Representative images of the liver of zebrafish. Scale bar = 50 μm. Boxplots show average size **(B)** and average number **(C)** of vacuoles in the liver of fish fed high cholesterol (HC) diet or HC diet supplemented with either algal glucan (AG) or oat glucan (OG) or simvastatin (SS), for a period of 12 weeks. Black dots inside each box indicate the mean values of the corresponding groups. * indicates *p* < 0.05 and • indicates *p* < 0.1. Each treatment group consisted of eight biological replicates.

**Figure 10:**
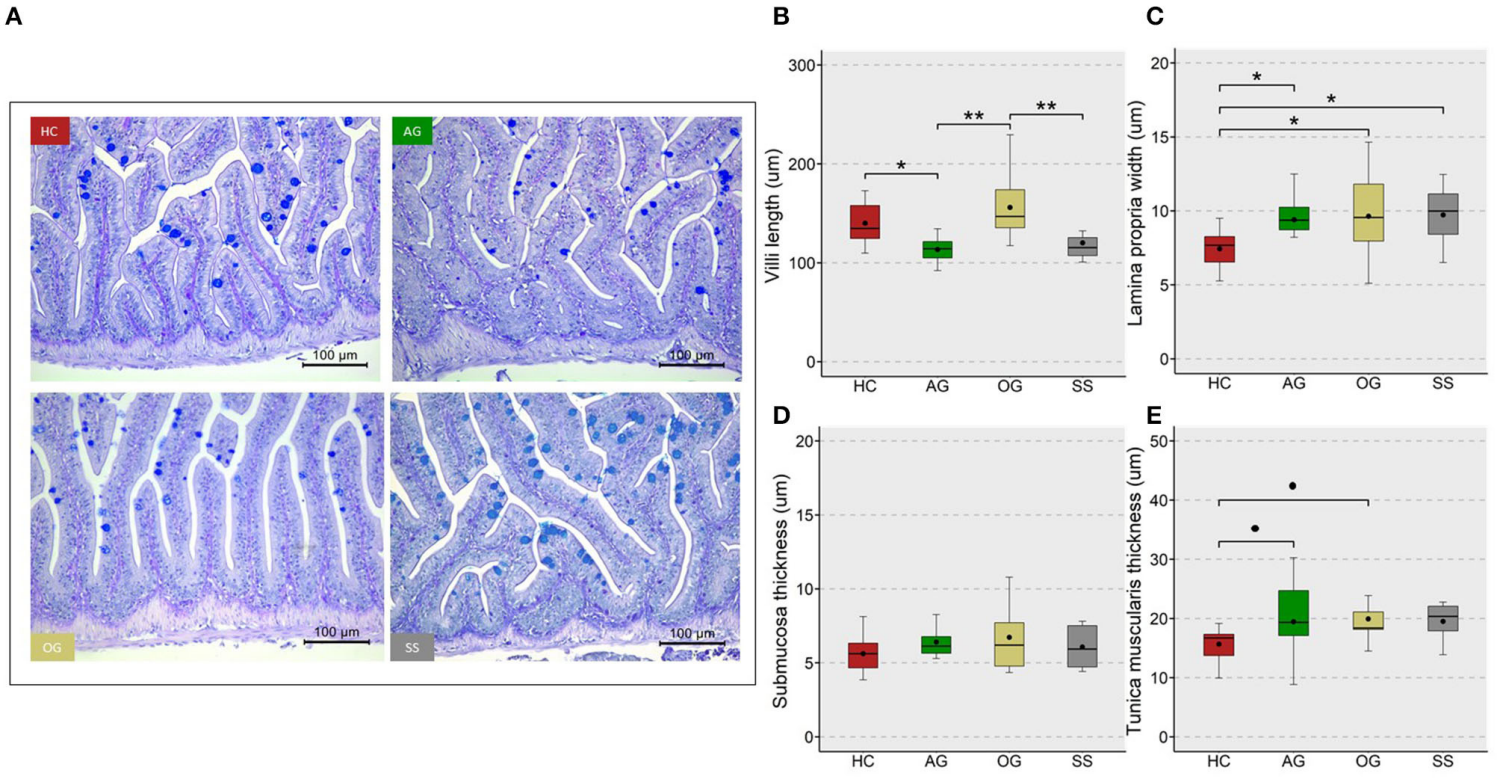
Micromorphology and feature indices of the intestine of the zebrafish. Representative images **(A)** from the different study groups. Scale bar = 100 μm. Boxplots show average villi length **(B)**, lamina propria width **(C)**, submucosa thickness **(D)** and tunica muscularis thickness **(E)** in the mid intestine of the fish fed experimental diets for a period of 12 weeks. Black dots inside each box indicate the mean values of the corresponding groups. ** indicates *p* < 0.01, * indicates *p* < 0.05, and • indicates *p* < 0.1. Each treatment group consisted of eight biological replicates.

We assessed the changes in hepatocyte vacuoles to understand the probable consequence of plasma hyperlipidemia; by measuring the average size and number of vacuoles in a selected area of the liver. We did not observe a significant increase in either the size or number of vacuoles in the fish fed high cholesterol diet (HC) compared to the CT diet (Supplementary Figure 7). On the other hand, the HC diet group had significantly larger vacuole area in their liver compared to the OG, AG and SS groups. Also, a trend for a lower number of vacuoles was observed in the OG (*p* = 0.07) and SS (*p* = 0.08) groups compared to the HC group (Figure 9). Fish fed the HC diet had longer intestinal villi compared to the CT group (Supplementary Figure 8). Furthermore, the algal glucan fed group had shorter villi compared to the HC and OG groups. The HC diet also significantly reduced the lamina propria thickness compared to the other groups and tunica muscularis thickness compared to glucan fed groups (Figure 10).

### Verification of the Expression of Selected Genes From the Transcriptomic Data

**Figure 11:**
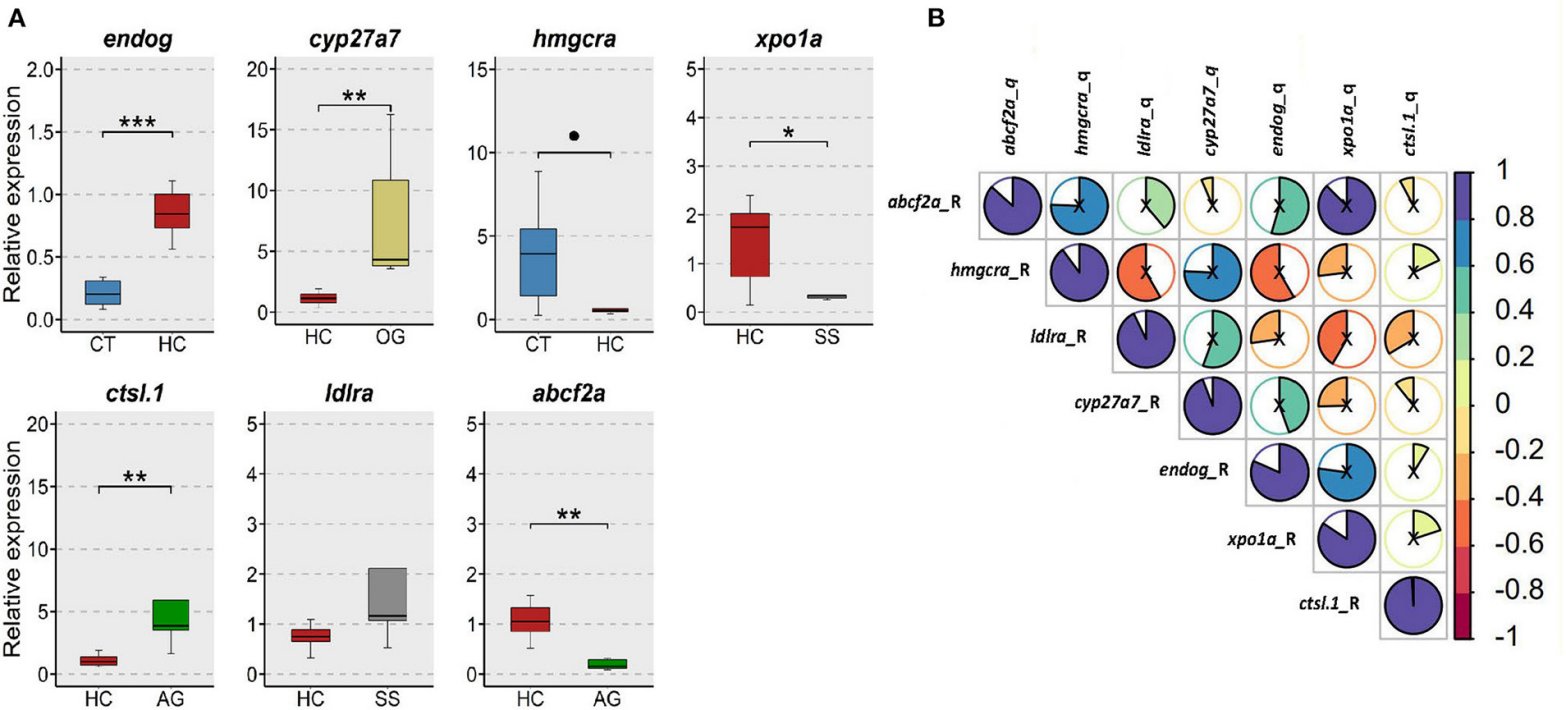
Verification of the expression of selected genes from the transcriptomic data. **(A)** Relative expression of 7 selected genes in the intestine of the zebrafish. CT, fish fed low cholesterol; HC, fish fed high cholesterol; AG, fish fed algal glucan; OG, fish fed oat glucan; SS, fish fed simvastatin. *n* = 5. *** indicate *p* < 0.001, ** indicates *p* < 0.01, * indicates *p* < 0.05, and • indicates *p* < 0.1. Each treatment group consisted of five biological replicates. **(B)** Correlation between the normalized counts from the RNA-Seq data and relative gene expression from the qPCR data.

From our RNA-Seq data, we selected 7 differentially expressed genes from different group comparisons for verification by qPCR (Figure 11A). These genes have critical functions in maintaining cholesterol homeostasis through regulation of cholesterol and bile acid biosynthesis (*hmgcra, abcf2a, cyp27a7*), vacuole formation (*ctsl.1*), protein synthesis (*xpo1a*), molecular chaperoning (*endog*) and lipoprotein (*ldlra*) receptor expression. Overall, the alterations in the expression of the selected genes agreed with the changes in the transcriptomic data (Figure 11B).

## Discussion

Consumption of a fiber-rich diet can be considered a healthy approach to prevent many diseases, including cancer, obesity, and hypercholesterolemia (49–51). In contrast to cholesterol-lowering drugs, β-glucans, the dietary fibers with bioactive properties, can positively impact lipid metabolism without exerting any side effects (52). Hence, in the present study, we employed two types of β-glucans; one derived from oats and the other from the alga, *P. tricornutum*. The rationale behind using β-glucans from different sources was to understand their distinct ability to lower cholesterol levels; due to their differential molecular structure, solubility, varying mode of action such as their gel-forming ability, prebiotic nature, effect on bile acids and microbiota. The synergy of β-glucans with their residues, as well as with the components in the food matrix also determines their bioactivity. In fact, for many decades β-glucans were thought to mitigate hypercholesterolemia through their ability to both increase the viscosity of the luminal contents and bind to bile acids (53), a property dependent on the branching pattern of β-glucans. Studies have revealed that consumption of 3 grams of β-glucans per day can mitigate hypercholesterolemia (28). There are certain products with proven cholesterol-lowering potential, e.g., PromOat® (Lantmännen Oats AB, Kimstad, Sweden) and OatWell™ (CreaNutrition, Lutterworth, UK) with European Food Safety Authority (EFSA) certificates; they are marketed in Europe, and the companies tout about the gel-forming ability of their products. On the other hand, a number of supplements for example EcoGard® (EderaGen Helse, Bergen, Norway) composed of fungal β-1,3 / 1,6-glucan and BioGlena™ (Algatechnologies Inc. Eilot, Israel) composed of *Euglena gracilis* β-1,3-glucan, are marketed as immunomodulators. We have used β-glucans from the microalga *P. tricornutum* and PromOat® in the present study. It is now well known that the effects of dietary β-glucans go beyond the lumen of the intestine i.e., they can alter the expression of lipid metabolism related genes of the intestinal cells (54). The cellular and molecular effects of β-glucans depend on their interaction with the cells via their specific receptors and signaling pathways. This interaction is dependent on the structural features making the source of β-glucans a vital factor that defines the hypocholesterolemic potential. In this context, the differential efficacy of β-glucans from different natural sources in alleviating hypercholesterolemia should be investigated because the polysaccharides may impact the host organs in multiple ways and with varying efficacies. In the marine diatom *P. tricornutum*, the main β-glucan form is chrysolaminarin, which consists of a linear β-1,3-glucan chain with limited β-1,6-glucan branching. β-glucans from oat comprise mainly β-D-glucopyranosyl monomers connected by either β-1,3 glucosidic bonds and/or β-1,4 glucosidic bonds. Although not measured in the present study, other researchers have indicated that chrysolaminarin has a low molecular weight, ranging between 1 and 20 kDa (55, 56), while oat beta-glucan has a high molecular weight, around 2,000 kDa (57). We compared these two sources of β-glucans–terrestrial plant (oat) and aquatic plant (microalga)–to enhance our understanding of the source-specific responses in the intestine, a crucial organ that regulates cholesterol metabolism. The plasma profile of zebrafish fed the two sources of glucans revealed that they are equally effective in mitigating hypercholesterolemia. The transcriptome comparisons revealed the plausible differential mode of action exerted by oat and microalga β-glucans to nullify the effects of hypercholesterolemia.

### Cholesterol in Diet Altered the Plasma Cholesterol Profile and Cholesterol Synthesis and Affected Important Organelle Functions

In our study, plasma total cholesterol and LDL-cholesterol were higher in adult zebrafish fed the HC diet. Previous studies have also reported that a high cholesterol diet can increase plasma cholesterol levels in adult zebrafish (32, 33). The rise in plasma cholesterol level induced by the high dietary cholesterol could be indicative of increased cholesterol absorption in the intestine or reduced clearance by the liver of the fish. The latter is less likely to be the case because there was no significant change in vacuolization in the liver of fish from the HC group compared to the CT group. *Niemann–Pick type C1-like 1* (*NPC1L1*) receptor, present on the apical surfaces of the intestinal epithelial cells, is an important transporter that facilitates the absorption of diet-derived free cholesterol via enterocytes. The expression of the *Npc1l1* gene in the intestine of mice was reduced by cholesterol feeding to maintain homeostasis of cholesterol metabolism (8). However, in our study *npc1l1* gene did not respond to 5.1% dietary cholesterol treatment. Another study that employed 3% purified cholesterol also did not report any change in the expression of *npc1l1* in the zebrafish intestine (58). This indicates that a homeostasis maintenance to regulate the absorption of cholesterol through the downregulation of *npc1l1* gene expression is probably missing in zebrafish intestine. This lack of homeostasis-maintenance mechanism directed toward cholesterol metabolism (59) makes zebrafish a unique model to recapitulate dyslipidemia without any genetic interventions.

Pathway analysis of the differentially expressed genes revealed that high dietary cholesterol levels can suppress *de novo* synthesis of cholesterol and bile (biosynthesis of steroid/terpenoid/bile acids) in the intestine, probably due to its increased absorption. Under normal circumstances, suppression of endogenous biosynthesis occurs because cholesterol biosynthesis requires significant inputs such as acetyl-CoA, ATP, oxygen and the reducing factors NADPH and NADH (60). Seven genes that are involved in cholesterol biosynthesis were downregulated in the HC diet fed fish, and this included *3-hydroxy-3-methylglutaryl-CoA reductase a* gene (*hmgcra*) that is involved in the formation of mevalonate, a rate-limiting step in cholesterol synthesis. These results probably indicate that zebrafish intestinal cholesterol metabolism is responsive to dietary cholesterol. The high intracellular cholesterol levels are likely to alter the functions of organelles such as ER and mitochondria because the upregulated genes from the HC vs. CT comparison were associated with the GO terms linked to ER and mitochondrial inner membrane and mitochondrial transport. It is known that cholesterol alters the inner mitochondrial membrane, thereby affecting its microviscosity (61, 62). Moreover, high intracellular cholesterol levels also impact the glutathione influx into the mitochondria, thus leading to a higher accumulation of the reactive oxygen species (ROS). The GO terms mitochondrial inner membrane and mitochondrial transport were enriched because of the upregulation of the genes *endonuclease G* (*endog*), *translocase of inner mitochondrial membrane 50 homolog (S. cerevisiae)* (*timm50*), *transmembrane protein 70* (*tmem70*), *solute carrier family 25 member 25b* (*slc25a25b*), *solute carrier family 25 member 38b* (*slc25a38b*) and *translocase of inner mitochondrial membrane 10 homolog (yeast)* (*timm10*). The gene *endog* is an important regulator of oxidative stress-mediated apoptosis. Exposure to ROS-producing agents is also known to induce the expression of *Endog* in rats (63). The *slc25a38b* gene codes for a protein that interacts with mitochondrial outer-membrane fusion proteins and maintains mitochondrial morphology (64). On the other hand the protein coded by *slc25a25b* is a calcium-binding molecule that transports nucleotides and cofactors across the mitochondria (65) and is involved in mitochondrial homeostasis. Altered expression of *Slc25a25* in mice was implicated in the resistance to diet-induced dysregulation of lipid metabolism (66). Cholesterol may also disrupt the assembly of the respiratory supracomplexes in the mitochondria (67). The genes *tmem70, timm50* and *timm10* are involved in the transport of different proteins and biogenesis of supracomplexes in the mitochondria (68, 69). We also observed dietary cholesterol-induced upregulation of the phagosome pathway in the HC group. The protein coded by the *SEC61 translocon gamma subunit* (*sec61*) gene normally transports proteins from the cytosol to the ER, but it can also reverse transport proteins from the ER to the cytosol for degradation (70). It is known that at least a subset of ER proteins contributes to the phagosome pathway (71). Furthermore, *sec61* is involved in transferring membrane protein from the ER to proteasome for destruction (72). These results indicate the impact of dietary cholesterol on ER, mitochondria, lipid metabolism and protein misfolding.

### Algal β-Glucan Impacted Certain Genes Connected to Lipid Metabolism and Cholesterol Efflux in the Intestine

Developing diet-based mitigation strategies against CVDs requires an understanding of the impact of therapeutic agents on circulating LDL-cholesterol levels. The reduction of the circulating total and LDL-cholesterol by dietary algal glucan was comparable to the protective effect provided by oat glucan and simvastatin. Although the comparison of the intestine transcriptome of the AG group with that of the HC group did not reveal precise pathways that explain the hypocholesterolemic effect of the algal glucan, the downregulation of key genes linked to lipid metabolism and vacuole formation could be informing the involvement of the molecules in lipid processing in the intestine tissue. Algal glucan possibly activated the processes linked to the GO term lysosome, as inferred from the upregulation of the genes *ifi30, ctsbb* and *ctsl.1*. Lysosomes are intracellular membrane-bound organelles characterized by an acidic pH and they contain a variety of hydrolytic enzymes. Cathepsin L1 (Ctsl1) and Cathepsin B (Ctsbb) are the most abundant lysosomal proteases and they participate in autophagy (73). Recognition of β-glucan by the membrane-associated receptors leads to lysosome activation through the unconventional vesicle-mediated secretion (74).

We observed downregulation of *keratin 15* (*krt15*) in the intestine of the algal glucan fed fish. The protein encoded by *krt15* is responsible for the structural integrity of mucosa, and it is expressed in the intestinal crypts and villi (75). The human keratin 14 gene is responsive to cellular levels of cholesterol the depletion of which can downregulate the gene (76). The expression of the gene *krt15* in our study likely indicates a response to reduced intracellular cholesterol. We also found a marked downregulation of the *cyp4v8* gene in the intestine of the alga glucan fed fish. Zebrafish have 94 *cyp* genes, which perform diverse functions; the *cyp4* clan contains four genes, including *cyp4v8*. Its ortholog in humans, *CYP4V2*, has been associated with the formation of omega-hydroxylated products (77). Activation of the lysosome pathway and the downregulation of *cyp4v8* in zebrafish fed algal glucan diet could be indicating an alteration in cholesterol transport because disturbances in the human ortholog is linked to lysosomal cholesterol transport (78). Another important downregulated gene in the AG group was *abcf2a*. ATP-binding cassette proteins are involved in the efflux of cholesterol from the intestine back to the lumen (79, 80). Western high energy diets are known to alter this gene, the expression of which is highly cell specific; while the *Abcf2* gene was upregulated in the endothelium, its expression in the parenchymal and Kupffer cells of the liver was not changed by the diet (81). Although the precise function of zebrafish *abcf2a* is not clearly described, its reduced expression indicates that this paralogue may be involved in the basolateral efflux of cholesterol in the intestine (82). Overall, the downregulation of *abcf2a, cyp4v8* and *krt15* genes indicate a possible effect of the microalgal β-glucan on the intestinal cholesterol metabolism and the efflux of cholesterol in the zebrafish model.

### Oat Beta Glucan Enhanced Lipid Catabolism in the Intestine

Dietary oat glucan specifically impacted the lipid metabolism of the intestinal tissue, and most of the GO terms indicated a link to lipid catabolism. The activation of the primary bile acid synthesis pathway in the intestine points towards the conversion of cholesterol to bile acids. Oat β-glucan is known to increase bile excretion, necessitating the *de novo* synthesis of bile (28). We found an indication of peroxisome activation, probably because of the need for certain enzymes present in the peroxisome (83). The upregulation of *cytochrome P450, family 27, subfamily A, polypeptide 1, gene 2* (*cyp27a1.2*) and *cytochrome P450, family 27, subfamily A, polypeptide 7* (*cyp27a7*) genes are linked to the conversion of cholesterol to bile. We detected the enrichment of PPAR signaling pathway because of the upregulation of, among others, the *acyl-CoA dehydrogenase medium chain* (*acadm*) gene that is involved in the breakdown of medium-chain fatty acids in the mitochondria.

### Simvastatin Altered Protein Metabolism in the Intestine

Simvastatin is a widely accepted cholesterol-lowering drug. Downregulation of genes and suppression of GO terms linked to ER evoked by both simvastatin and β-glucans validate the ability of glucans to mitigate the effects of high dietary cholesterol in the zebrafish model. Although statins are considered safe hypocholesterolemic drugs for humans, they are often associated with statin-associated skeletal muscle problems (84, 85). The protein synthesis machinery (86) can be impaired, resulting in statin-induced myopathy. Such effects have been reported in zebrafish as well (87). However, we are the first to report simvastatin-caused transcriptomic responses in the intestine of a vertebrate model organism. Based on our results, dietary simvastatin suppressed translation, rRNA modification, rRNA metabolic process and peptide biosynthetic process indicating an impact of the product on the protein synthesis machinery of the intestine. Statin-induced depletion of cholesterol in the striated muscle cells destabilizes membrane potential and alters ion balance in the cells thus affecting protein synthesis (88). Suppression of cholesterol biosynthesis by statins also leads to the deficiency of intermediates like farnesyl pyrophosphate and ubiquinone which are generated during cholesterol biosynthesis. These intermediates are important for sarcoplasm and mitochondrial functions and their unavailability may trigger myopathy (89). Prior studies have also revealed the suppressive effect of cholesterol-lowering drugs on protein synthesis (90). Although simvastatin at 50 mg kg^−1^ of diet revealed an effect on the protein synthesis in the intestine, the impact on the tunica muscularis thickness in the zebrafish intestine was not evident in the present study. Akin to the observations in the fish fed with algal glucan, simvastatin also led to lysosomal activation. Likewise, ER-related processes were suppressed by both oat glucan and simvastatin.

### Adverse Effects of Dietary Cholesterol Were Alleviated by β-Glucans and Simvastatin

The ER membrane holds many enzymes associated with cholesterol metabolism. High cholesterol-induced misfolding of proteins influences ER homeostasis. Several GO terms like endoplasmic reticulum membrane, cellular response to topologically incorrect protein, and cellular response to unfolded protein were enriched in the HC diet group. It has been proposed that cholesterol-induced dysfunction of the ER calcium pumps affects the calcium-dependent chaperones and consequently ER protein folding (91). The subsequent ER stress causes the progression of cardiovascular diseases (92). Our study revealed that all three treatments–algal glucan, oat glucan, and simvastatin–can alleviate the adverse effects on ER in the intestine of the zebrafish. Although the AG vs. HC comparison did not reveal any suppression of GO terms or pathways related to ER stress, we observed the downregulation of the gene *calua* in fish fed the algal glucan. This gene codes for an ER chaperone protein, a potent suppressor of ER stress mediated signaling cascade and is considered a marker of ER stress. The downregulation of *calua* expression has also been associated with the attenuation of ER stress (93). Therefore, we speculate that algal glucan can also relieve the intestinal tissue from cholesterol-induced ER stress. Oat glucan and simvastatin, on the other hand, reduced the expression of several heat shock proteins (HSPs) in the intestine. HSPs comprise a group of highly conserved, ubiquitous molecules, promoting proper folding and assembling of polypeptides. An important aspect of unfolded protein response is the upregulation of HSP genes (94, 95). Expression of HSP genes like *heat shock protein 5* (*hspa5*), *heat shock protein 90, beta (grp94), member 1* (*hsp90b1*), *hypoxia up-regulated 1* (*hyou1*) and *heat shock cognate 70-kd protein, tandem duplicate 3* (*hsp70.3*) was downregulated in the intestine of zebrafish fed oat glucan and simvastatin. We speculate that the observed downregulation of molecular chaperone genes that code for HSPs and Calua is an indication of reduced ER stress.

The distinct alterations in the intestinal transcripts evoked by algal and oat glucans have indicated the possible synergistic actions that could be exploited to counter hypercholesterolemia (Supplementary Figure 9). Our results indicate that algal glucans are effective against hypercholesterolemia, possibly through the downregulation of the cholesterol transporter gene *abcf2a* and the cytochrome P450 family gene, *cyp4v8*. Reduced expression of the *cyp4v8* ortholog in humans is known to increase the free cholesterol content in cells (78), whereas alterations in the expression of *abcf2a* gene could suppress basolateral transport of free cholesterol in enterocytes (82). Overall, the alterations in the expression *abcf2a* and *cyp4v8* genes indicate a possible accumulation of free cholesterol in the enterocytes of fish fed the AG diet. On the other hand, in the OG group, we found an increased steroid hydroxylase activity and primary bile acid biosynthesis activity. This indicates a compensatory mechanism of *de novo* bile synthesis possibly activated in response to reduced bile absorption in the intestine stimulated by oat glucans (28, 96). A synergy between the two glucans could activate their specific mechanisms, i.e., reduced cholesterol transport and reduced bile absorption to eventually reduce the circulating cholesterol levels. It could be hypothesized that the synergistic cholesterol-lowering effect of the two glucans can further reduce the dependence on the statins.

### Cholesterol and β-Glucans Affected Vacuolization in the Liver and Micromorphology of the Intestine

Zebrafish is an excellent model for studying liver diseases because of the homology of the organ system compared to humans. Although zebrafish has a unique hepatic anatomy, ongoing research has revealed the conserved cell populations, transcriptional profile and signaling pathways associated with zebrafish and the human liver (97). Moreover, the cells in the zebrafish liver are involved in cholesterol metabolism and lipid storage (98). Therefore, zebrafish has been used to study fatty liver disease and is considered a model species to investigate liver metabolic dysfunctions. As for the extent of vacuolization in the liver of fish from different groups, although the mean number of vacuoles was higher in the HC compared to the CT group, we did not observe any significant differences. We speculate that the non-significant difference in vacuolization is linked to the age of the experimental fish and the dietary lipid levels. One-year-old adult fish were chosen for two purposes: to retrieve enough plasma for biochemical analyses and because they can be employed to mimic human diet-induced dyslipidemia. A high vacuolization tendency has been observed in the liver of 16 to 22-month-old zebrafish fed 5% dietary lipid (99). Furthermore, lipid and glycogen vacuolization has also been observed in previous studies wherein zebrafish were fed 11 to 13% lipids (100, 101). The low cholesterol diet in our study with 12% lipid also led to lipid accumulation in the liver of zebrafish. Such lipid vacuolization is likely due to the perturbed energy homeostasis that was induced by formulated feeds (102). Unfortunately, the optimum dietary lipid requirement of zebrafish has not been investigated in detail (103, 104).

β-glucans and simvastatin were able to reduce the vacuolization in the liver of zebrafish. The ability of β-glucan and simvastatin to mitigate fat deposition in the liver has been reported earlier (105, 106). Our findings indicate that β-glucans have a hypolipidemic effect that extends beyond the intestinal tissue. We also found significant changes in the histology of the mid intestine. A general increase in villi length and thinning of the lamina propria was observed in the HC group compared to the CT group. Like in mammals, the zebrafish villi length is regulated by stem cell proliferation at the base and apoptosis at the tip of the villi. Dysregulated lipid metabolism leading to obesity increases the villi length in humans (107). Suppression of endogenous cholesterol biosynthesis (108) and higher intracellular levels of cholesterol (109) induce hypertrophy in different types of cells. Cholesterol acts as a mitogen for intestinal stem cells, and an increase in cellular cholesterol content activates stem cell proliferation both *in vivo* and *ex vivo* (110). Therefore, dietary cholesterol may have increased the stem cell proliferation in the mid intestine leading to the observed increase in the villi length and an associated lamina propria thinning.

### Other Factors That May Impact the Lipid Metabolism and Efficacy of β-Glucans

In the present study, we did not investigate the changes in the intestinal microbiota of the fish. Nevertheless, it is known that gut microbiota regulates the total cholesterol, circulating LDL-cholesterol and triglyceride levels that are considered as risk factors of cardiovascular disease (111). β-glucans lower the LDL, total cholesterol and serum triglyceride levels through the proliferation of intestinal microbes that possess bile salt hydrolase (BSH) activity (112, 113). BSH activity can deconjugate bile acids, that are ligands for farnesoid X receptor (FXR), which is the intestinal bile acid sensor and the controller of liver bile acid production and lipid metabolism (114); this function of FXR is conserved in mammals and zebrafish (115). The positive effects of the β-glucan-driven increase in the BSH activity on gut physiology and lipid metabolism are not yet fully clarified.

The purity of β-glucans and feed manufacturing processes can also govern the efficacy of β-glucans to counter hypercholesterolemia. Several compounds other than β-glucans that are present in oats can modulate the metabolic activity in humans (116, 117). As for the extract from the microalga, *P. tricornutum*, functional carbohydrates other than glucans might have influenced the responses in zebrafish. In our study, the β-glucans from the two sources were 30% pure and therefore, we cannot rule out the contribution of other molecules in the test products. They might have acted synergistically to evoke the responses that we report here. Studies are being conducted to reduce the residues in β-glucans supplements (118). Furthermore, processing can modify the physicochemical characteristics such as molecular weight, extractability and the resulting viscosity (119). The low-shear extrusion process that was adopted to produce the zebrafish feeds employed a moderate processing temperature (50–60 °C) and fluidised air drying at 80 °C for approximately 10–12 min. Extrusion can alter the branching structure and hence reduce the molecular weight. Although β-glucans of high molecular weight are regarded as efficient in lowering the serum cholesterol and shaping the microbiota to reduce the risk of developing cardiovascular disease (120), the efficacy of β-glucans is dependent on many other factors e.g., residues in the product, molecular structure, viscosity, solubility and the surrounding food matrix (23, 26, 116). Future studies regarding the application of β-glucans should assess the effects of food processing variables on the structural integrity and impact on the biological activity of β-glucans.

In conclusion, our study showed that the intestine transcriptome of the zebrafish was highly sensitive to dietary cholesterol. Furthermore, endogenous cholesterol biosynthesis was suppressed, and ER and mitochondrial functions were affected by the dietary cholesterol. Our transcriptome analyses revealed the efficacy of β-glucans as well as simvastatin to counter hypercholesterolemia, by restoring the expression of several genes that were altered by dietary cholesterol. It seems that the three compounds work to reduce the stress on the ER. While the action of algal glucan is likely via lysosome and cholesterol efflux from the intestine, the oat glucan might have employed the cholesterol catabolism path to move to a normocholesterolemic condition. We also observed a significant adverse effect of simvastatin on the protein metabolism in the intestinal tissue. Future studies should explore the combinatorial effect of the two glucans or one of the glucans and the statin because the investigations will disclose new clues to their synergistic effect to tackle hypercholesterolemia.

## Data Availability Statement

The datasets presented in this study can be found in online repositories. The names of the repository/repositories and accession number(s) can be found in the article/Supplementary Material.

## Ethics Statement

The approval for the conduct of this study was obtained from the Norwegian Animal Research Authority (FDU ID: 22992).

## Author Contributions

VK, JD, AG, and MC: conceptualization and study design. AG, SR, and PS: feeding experiment and tissue sampling. AG and SR: library preparation and bioinformatic analysis. AG and IV: histological analysis. SR: qPCR analysis. AG: biochemical tests. AG and VK: manuscript writing. All authors: manuscript revisions.

## Funding

This work was funded by Nord University and has received partial funding from the Bio Based Industries Joint Undertaking (BBI JU) under the European Union's Horizon 2020 research and innovation programme under grant agreement no. 745754 (project MAGNIFICENT). The funder was not involved in the study design, collection, analysis, interpretation of data, the writing of this article or the decision to submit it for publication. This output reflects the views only of the author(s), and the European Union and BBI JU cannot be held responsible for any use which may be made of the information contained therein. SR and AG were supported by Netaji Subhas-ICAR International Fellowships (NS-ICAR IFs) from the Indian Council of Agricultural Research, India.

## Conflict of Interest

JD was employed by company SPAROS Lda. MC was employed by company MadeBiotech and NatureXtracts S.A. The remaining authors declare that the research was conducted in the absence of any commercial or financial relationships that could be construed as a potential conflict of interest.

## Publisher's Note

All claims expressed in this article are solely those of the authors and do not necessarily represent those of their affiliated organizations, or those of the publisher, the editors and the reviewers. Any product that may be evaluated in this article, or claim that may be made by its manufacturer, is not guaranteed or endorsed by the publisher.

## References

[1] Anand SS Hawkes C de Souza RJ Mente A Dehghan M Nugent R. Food consumption and its impact on cardiovascular disease: importance of solutions focused on the globalized food system: a report from the workshop convened by the world heart federation. J Am Coll Cardiol. (2015) 66:1590–614. 10.1016/j.jacc.2015.07.050 26429085 PMC4597475

[2] Vincent MJ Allen B Palacios OM Haber LT Maki KC. Meta-regression analysis of the effects of dietary cholesterol intake on LDL and HDL cholesterol. Am J Clin Nutr. (2019) 109:7–16. 10.1093/ajcn/nqy273 30596814

[3] Ference BA Ginsberg HN Graham I Ray KK Packard CJ Bruckert E. Low-density lipoproteins cause atherosclerotic cardiovascular disease. 1 evidence from genetic, epidemiologic, and clinical studies a consensus statement from the European atherosclerosis society consensus panel. Eur Heart J. (2017) 38:2459–72. 10.1093/eurheartj/ehx144 28444290 PMC5837225

[4] Zhang T Yuan D Xie J Lei Y Li J Fang G. Evolution of the cholesterol biosynthesis pathway in animals. Mol Biol Evol. (2019) 36:2548–56. 10.1093/molbev/msz167 31397867

[5] Gabbia D Roverso M Guido M Sacchi D Scaffidi M Carrara M. Western diet-induced metabolic alterations affect circulating markers of liver function before the development of steatosis. Nutrients. (2019) 11:1602. 10.3390/nu11071602 31311123 PMC6683046

[6] Borén J Chapman MJ Krauss RM Packard CJ Bentzon JF Binder CJ. Low-density lipoproteins cause atherosclerotic cardiovascular disease: pathophysiological, genetic, and therapeutic insights: a consensus statement from the European atherosclerosis society consensus panel. Eur Heart J. (2020) 41:2313–30. 10.1093/eurheartj/ehz962 32052833 PMC7308544

[7] Barter PJ Nicholls S Rye KA Anantharamaiah GM Navab M Fogelman AM. Antiinflammatory properties of HDL. Circ Res. (2004) 95:764–72. 10.1161/01.RES.0000146094.59640.13 15486323

[8] Davis HR Zhu LJ Hoos LM Tetzloff G Maguire M Liu J. Niemann-Pick C1 Like 1 (NPC1L1) is the intestinal phytosterol and cholesterol transporter and a key modulator of whole-body cholesterol homeostasis. J Biol Chem. (2004) 279:33586–92. 10.1074/jbc.M405817200 15173162

[9] Ticho AL Malhotra P Dudeja PK Gill RK Alrefai WA. Bile acid receptors and gastrointestinal functions. Liver Res. (2019) 3:31–9. 10.1016/j.livres.2019.01.001 32368358 PMC7197881

[10] Lo C-M Nordskog BK Nauli AM Zheng S vonLehmden SB Yang Q. Why does the gut choose apolipoprotein B48 but not B100 for chylomicron formation? Am J Physiol Gastrointest Liver Physiol. (2008) 294:G344–52. 10.1152/ajpgi.00123.2007 18006607

[11] Mansbach CM Siddiqi SA. The biogenesis of chylomicrons. Annu Rev Physiol. (2010) 72:315–33. 10.1146/annurev-physiol-021909-135801 20148678 PMC4861230

[12] Goldstein JL Brown MS. Regulation of low-density lipoprotein receptors: implications for pathogenesis and therapy of hypercholesterolemia and atherosclerosis. Circulation. (1987) 76:504–7. 10.1161/01.CIR.76.3.504 3621516

[13] Zhang L Reue K Fong LG Young SG Tontonoz P. Feedback regulation of cholesterol uptake by the LXR-IDOL-LDLR axis. Arterioscler Thromb Vasc Biol. (2012) 32:2541–6. 10.1161/ATVBAHA.112.250571 22936343 PMC4280256

[14] Benn M. Apolipoprotein B levels, APOB alleles, and risk of ischemic cardiovascular disease in the general population, a review. Atherosclerosis. (2009) 206:17–30. 10.1016/j.atherosclerosis.2009.01.004 19200547

[15] du Souich P Roederer G Dufour R. Myotoxicity of statins: mechanism of action. Pharmacol Ther. (2017) 175:1–16. 10.1016/j.pharmthera.2017.02.029 28223230

[16] Sathasivam S. Statin induced myotoxicity. Eur J Intern Med. (2012) 23:317–24. 10.1016/j.ejim.2012.01.004 22560377

[17] Godard M Décordé K Ventura E Soteras G Baccou J-C Cristol J-P. Polysaccharides from the green alga *Ulva rigida* improve the antioxidant status and prevent fatty streak lesions in the high cholesterol fed hamster, an animal model of nutritionally-induced atherosclerosis. Food Chem. (2009) 115:176–80. 10.1016/j.foodchem.2008.11.084

[18] Kimura Y Watanabe K Okuda H. Effects of soluble sodium alginate on cholesterol excretion and glucose tolerance in rats. J Ethnopharmacol. (1996) 54:47–54. 10.1016/0378-8741(96)01449-3 8941868

[19] Du B Lin C Bian Z Xu B. An insight into anti-inflammatory effects of fungal beta-glucans. Trends Food Sci Technol. (2015) 41:49–59. 10.1016/j.tifs.2014.09.002

[20] Henrion M Francey C Lê K-A Lamothe L. Cereal β-glucans: The impact of processing and how it affects physiological responses. Nutrients. (2019) 11:1729. 10.3390/nu11081729 31357461 PMC6722849

[21] Myklestad S. Production of carbohydrates by marine planktonic diatoms. I comparison of nine different species in culture. J Exp Mar Biol Ecol. (1974) 15:261–74. 10.1016/0022-0981(74)90049-5

[22] Chiovitti A Molino P Crawford SA Teng R Spurck T Wetherbee R. The glucans extracted with warm water from diatoms are mainly derived from intracellular chrysolaminaran and not extracellular polysaccharides. Eur J Phycol. (2004) 39:117–28. 10.1080/0967026042000201885

[23] Han B Baruah K Cox E Vanrompay D Bossier P. Structure-functional activity relationship of β-glucans from the perspective of immunomodulation: a mini-review. Front Immunol. (2020) 11:658. 10.3389/fimmu.2020.00658 32391005 PMC7188827

[24] Du B Meenu M Liu H Xu B. A concise review on the molecular structure and function relationship of β-glucan. Int J Mol Sci. (2019) 20:4032. 10.3390/ijms20164032 31426608 PMC6720260

[25] Adams EL Rice PJ Graves B Ensley HE Yu H Brown GD. Differential high-affinity interaction of dectin-1 with natural or synthetic glucans is dependent upon primary structure and is influenced by polymer chain length and side-chain branching. J Pharmacol Exp Ther. (2008) 325:115–23. 10.1124/jpet.107.133124 18171906

[26] Kim HJ White PJ. In vitro bile-acid binding and fermentation of high, medium, and low molecular weight β-glucan. J Agric Food Chem. (2010) 58:628–34. 10.1021/jf902508t 20020684

[27] Shoukat M Sorrentino A. Cereal β-glucan: a promising prebiotic polysaccharide and its impact on the gut health. Int J Food Sci. (2021) 56:2088–97. 10.1111/ijfs.1497133609583

[28] Joyce SA Kamil A Fleige L Gahan CGM. The cholesterol-lowering effect of oats and oat beta glucan: modes of action and potential role of bile acids and the microbiome. Front Nutr. (2019) 6:171. 10.3389/fnut.2019.00171 31828074 PMC6892284

[29] Kerckhoffs DA Hornstra G Mensink RP. Cholesterol-lowering effect of β-glucan from oat bran in mildly hypercholesterolemic subjects may decrease when β-glucan is incorporated into bread and cookies. Am J Clin Nutr. (2003) 78:221–7. 10.1093/ajcn/78.2.221 12885701

[30] Reyna-Villasmil N Bermúdez-Pirela V Mengual-Moreno E Arias N Cano-Ponce C Leal-Gonzalez E. Oat-derived beta-glucan significantly improves HDLC and diminishes LDLC and non-HDL cholesterol in overweight individuals with mild hypercholesterolemia. Am J Ther. (2007) 14:203–12. 10.1097/01.pap.0000249917.96509.e7 17414591

[31] Ka J Jin S-W. Zebrafish as an emerging model for dyslipidemia and associated diseases. J Lipid Atheroscler. (2021) 10:42–56. 10.12997/jla.2021.10.1.42 33537252 PMC7838516

[32] Stoletov K Fang L Choi S-H Hartvigsen K Hansen LF Hall C. Vascular lipid accumulation, lipoprotein oxidation, and macrophage lipid uptake in hypercholesterolemic zebrafish. Circ Res. (2009) 104:952–60. 10.1161/CIRCRESAHA.108.189803 19265037 PMC2834250

[33] Yoon Y Yoon J Jang MY Na Y Ko Y Choi JH. High cholesterol diet induces IL-1β expression in adult but not larval zebrafish. PLoS ONE. (2013) 8:e66970. 10.1371/journal.pone.0066970 23825600 PMC3692503

[34] Fang L Green SR Baek JS Lee SH Ellett F Deer E. In vivo visualization and attenuation of oxidized lipid accumulation in hypercholesterolemic zebrafish. J Clin Invest. (2011) 121:4861–9. 10.1172/JCI57755 22105168 PMC3225997

[35] Ko C-W Qu J Black DD Tso P. Regulation of intestinal lipid metabolism: current concepts and relevance to disease. Nat Rev Gastroenterol Hepatol. (2020) 17:169–83. 10.1038/s41575-019-0250-7 32015520

[36] Westerfield M. The Zebrafish Book: A Guide For The Laboratory Use Of Zebrafish. (2000). Available online at: http://zfin.org/zf_info/zfbook/zfbk.html

[37] Dai W Wang K Zheng X Chen X Zhang W Zhang Y. High fat plus high cholesterol diet lead to hepatic steatosis in zebrafish larvae: a novel model for screening anti-hepatic steatosis drugs. Nutr Metab. (2015) 12:42. 10.1186/s12986-015-0036-z 26583037 PMC4650307

[38] Babaei F Ramalingam R Tavendale A Liang Y Yan LSK Ajuh P. Novel blood collection method allows plasma proteome analysis from single zebrafish. J Proteome Res. (2013) 12:1580–90. 10.1021/pr3009226 23413775

[39] Siriyappagouder P Galindo-Villegas J Dhanasiri AKS Zhang Q Mulero V Kiron V et al. *Pseudozyma* priming influences expression of genes involved in metabolic pathways and immunity in zebrafish larvae. Front Immunol. (2020) 11:978. 10.3389/fimmu.2020.00978 32528473 PMC7256946

[40] Chen S Zhou Y Chen Y Gu J. fastp: an ultra-fast all-in-one FASTQ preprocessor. Bioinformatics. (2018) 34:884–90. 10.1093/bioinformatics/bty560 30423086 PMC6129281

[41] Kim D Langmead B Salzberg SL. HISAT: a fast spliced aligner with low memory requirements. Nat Methods. (2015) 12:357–60. 10.1038/nmeth.3317 25751142 PMC4655817

[42] Liao Y Smyth GK Shi W. FeatureCounts: an efficient general purpose program for assigning sequence reads to genomic features. Bioinformatics. (2014) 30:923–30. 10.1093/bioinformatics/btt656 24227677

[43] Shannon P Markiel A Ozier O Baliga NS Wang JT Ramage D. Cytoscape: a software environment for integrated models of biomolecular interaction networks. Genome Res. (2003) 13:2498–504. 10.1101/gr.1239303 14597658 PMC403769

[44] Supek F Bošnjak M Škunca N Šmuc T. REVIGO summarizes and visualizes long lists of gene ontology terms. PLoS ONE. (2011) 6:e21800. 10.1371/journal.pone.0021800 21789182 PMC3138752

[45] Bancroft JD Gamble M. Theory And Practice Of Histological Techniques. London: Churchill Livingstone (2008).

[46] Schneider CA Rasband WS Eliceiri KW. NIH image to imageJ: 25 years of image analysis. Nat Methods. (2012) 9:671–5. 10.1038/nmeth.2089 22930834 PMC5554542

[47] Rehman S Gora AH Siriyappagouder P Brugman S Fernandes JMO Dias J. Zebrafish intestinal transcriptome highlights subdued inflammatory responses to dietary soya bean and efficacy of yeast β-glucan. J Fish Dis. (2021) 44:1619–37. 10.1111/jfd.13484 34237181

[48] Tang R Dodd A Lai D McNabb WC Love DR. Validation of zebrafish (*Danio rerio*) reference genes for quantitative real-time RT-PCR normalization. Acta Biochim Biophys Sin. (2007) 39:384–90. 10.1111/j.1745-7270.2007.00283.x 17492136 PMC7110012

[49] Nie Y Luo F. Dietary fiber: an opportunity for a global control of hyperlipidemia. Oxid Med Cell Longev. (2021) 2021:5542342. 10.1155/2021/5542342 33897940 PMC8052145

[50] Hullings AG Sinha R Liao LM Freedman ND Graubard BI Loftfield E. Whole grain and dietary fiber intake and risk of colorectal cancer in the NIH-AARP diet and health study cohort. Am J Clin Nutr. (2020) 112:603–12. 10.1093/ajcn/nqaa161 32619213 PMC7458778

[51] Mayengbam S Lambert JE Parnell JA Tunnicliffe JM Nicolucci AC Han J. Impact of dietary fiber supplementation on modulating microbiota–host–metabolic axes in obesity. J Nutr Biochem. (2019) 64:228–36. 10.1016/j.jnutbio.2018.11.003 30572270

[52] Thompson PD Panza G Zaleski A Taylor B. Statin-associated side effects. J Am Coll Cardiol. (2016) 67:2395–410. 10.1016/j.jacc.2016.02.071 27199064

[53] Chau CF Huang YL. Effects of the insoluble fiber derived from passiflora edulis seed on plasma and hepatic lipids and fecal output. Mol Nutr Food Res. (2005) 49:786–90. 10.1002/mnfr.200500060 15995986

[54] Drozdowski LA Reimer RA Temelli F Bell RC Vasanthan T Thomson AB. Beta-glucan extracts inhibit the in vitro intestinal uptake of long-chain fatty acids and cholesterol and down-regulate genes involved in lipogenesis and lipid transport in rats. J Nutr Biochem. (2010) 21:695–701. 10.1016/j.jnutbio.2009.04.003 19716281 PMC3833848

[55] Paulsen BS Myklestad S. Structural studies of the reserve glucan produced by the marine diatom *Skeletonema costatum* (grev) Cleve. Carbohydr Res. (1978) 62:386–8. 10.1016/S0008-6215(00)80888-5

[56] Chiovitti A Higgins MJ Harper RE Wetherbee R Bacic A. The complex polysaccharides of the raphid diatom *Pinnularia viridis* (Bacillariophyceae). J Phycol. (2003) 39:543–54. 10.1046/j.1529-8817.2003.02162.x

[57] Immerstrand T Andersson KE Wange C Rascon A Hellstrand P Nyman M. Effects of oat bran, processed to different molecular weights of β-glucan, on plasma lipids and caecal formation of SCFA in mice. Br J Nutr. (2010) 104:364–73. 10.1017/S0007114510000553 20334710

[58] Takase M Ushio H. Changes in intestinal gene expression of zebrafish (*Danio rerio*) related to sterol uptake and excretion upon β-sitosterol administration. Fishes. (2018) 3:1. 10.3390/fishes301000129683143

[59] Schlegel A. Zebrafish models for dyslipidemia and atherosclerosis research. Front Endocrinol. (2016) 7:159. 10.3389/fendo.2016.00159 28018294 PMC5159437

[60] Cerqueira NMFSA Oliveira EF Gesto DS Santos-Martins D Moreira C Moorthy HN. Cholesterol biosynthesis: a mechanistic overview. Biochemistry. (2016) 55:5483–506. 10.1021/acs.biochem.6b00342 27604037

[61] Montero J Mari M Colell A Morales A Basañez G Garcia-Ruiz C. Cholesterol and peroxidized cardiolipin in mitochondrial membrane properties, permeabilization and cell death. Biochim Biophys Acta. (2010) 1797:1217–24. 10.1016/j.bbabio.2010.02.010 20153716 PMC2889134

[62] Bosch M Marí M Herms A Fernández A Fajardo A Kassan A. Caveolin-1 deficiency causes cholesterol-dependent mitochondrial dysfunction and apoptotic susceptibility. Curr Biol. (2011) 21:681–6. 10.1016/j.cub.2011.03.030 21497090 PMC3409647

[63] Lee JS Seo TW Yi JH Shin KS Yoo SJ. CHIP has a protective role against oxidative stress-induced cell death through specific regulation of endonuclease G. Cell Death Dis. (2013) 4:e666–e666. 10.1038/cddis.2013.181 23764847 PMC3698548

[64] Zhang C Shi Z Zhang L Zhou Z Zheng X Liu G. Appoptosin interacts with mitochondrial outer-membrane fusion proteins and regulates mitochondrial morphology. J Cell Sci. (2016) 129:994–1002. 10.1242/jcs.176792 26813789 PMC4813315

[65] Amigo I Traba J González-Barroso MM Rueda CB Fernández M Rial E. Glucagon regulation of oxidative phosphorylation requires an increase in matrix adenine nucleotide content through Ca2^+^ activation of the mitochondrial ATP-Mg/Pi carrier SCaMC-3. J Biol Chem. (2013) 288:7791–802. 10.1074/jbc.M112.409144 23344948 PMC3597818

[66] Anunciado-Koza RP Zhang J Ukropec J Bajpeyi S Koza RA Rogers RC. Inactivation of the mitochondrial carrier SLC25A25 (ATP-Mg2^+^/Pi transporter) reduces physical endurance and metabolic efficiency in mice. J Biol Chem. (2011) 286:11659–71. 10.1074/jbc.M110.203000 21296886 PMC3064218

[67] Solsona-Vilarrasa E Fucho R Torres S Nuñez S Nuño-Lámbarri N Enrich C. Cholesterol enrichment in liver mitochondria impairs oxidative phosphorylation and disrupts the assembly of respiratory supercomplexes. Redox Biol. (2019) 24:101214. 10.1016/j.redox.2019.101214 31108462 PMC6526464

[68] Sánchez-Caballero L Elurbe DM Baertling F Guerrero-Castillo S van den Brand M van Strien J. TMEM70 functions in the assembly of complexes I and V. Biochim Biophys Acta Bioenerg. (2020) 1861:148202. 10.1016/j.bbabio.2020.148202 32275929

[69] Heinemeyer T Stemmet M Bardien S Neethling A. Underappreciated roles of the translocase of the outer and inner mitochondrial membrane protein complexes in human disease. DNA Cell Biol. (2019) 38:23–40. 10.1089/dna.2018.4292 30481057

[70] Ackerman AL Giodini A Cresswell P. A role for the endoplasmic reticulum protein retrotranslocation machinery during crosspresentation by dendritic cells. Immunity. (2006) 25:607–17. 10.1016/j.immuni.2006.08.017 17027300

[71] Campbell-Valois F-X Trost M Chemali M Dill BD Laplante A Duclos S. Quantitative proteomics reveals that only a subset of the endoplasmic reticulum contributes to the phagosome. Mol Cell Proteomics. (2012) 11:M111.016378-1–13. 10.1074/mcp.M111.016378 22427703 PMC3394953

[72] Wiertz EJ Tortorella D Bogyo M Yu J Mothes W Jones TR. Sec61-mediated transfer of a membrane protein from the endoplasmic reticulum to the proteasome for destruction. Nature. (1996) 384:432–8. 10.1038/384432a0 8945469

[73] Kaminskyy V Zhivotovsky B. Proteases in autophagy. Biochim Biophys Acta. (2012) 1824:44–50. 10.1016/j.bbapap.2011.05.013 21640203

[74] Öhman T Teirilä L Lahesmaa-Korpinen A-M Cypryk W Veckman V Saijo S. Dectin-1 pathway activates robust autophagy-dependent unconventional protein secretion in human macrophages. J Immunol. (2014) 192:5952. 10.4049/jimmunol.1303213 24808366

[75] Giroux V Stephan J Chatterji P Rhoades B Wileyto EP Klein-Szanto AJ. Mouse intestinal Krt15+ crypt cells are radio-resistant and tumor initiating. Stem cell reports. (2018) 10:1947–58. 10.1016/j.stemcr.2018.04.022 29805107 PMC5993649

[76] Jans R Atanasova G Jadot M Poumay Y. Cholesterol depletion upregulates involucrin expression in epidermal keratinocytes through activation of p38. J Invest Dermatol. (2004) 123:564–73. 10.1111/j.0022-202X.2004.23221.x 15304097

[77] Nakano M Kelly EJ Rettie AE. Expression and characterization of CYP4V2 as a fatty acid omega-hydroxylase. Drug Metab Dispos. (2009) 37:2119–22. 10.1124/dmd.109.028530 19661213 PMC2774980

[78] Hata M Ikeda HO Iwai S Iida Y Gotoh N Asaka I. Reduction of lipid accumulation rescues Bietti's crystalline dystrophy phenotypes. Proc Natl Acad Sci U S A. (2018) 115:3936–41. 10.1073/pnas.1717338115 29581279 PMC5899444

[79] Temel RE Lee RG Kelley KL Davis MA Shah R Sawyer JK. Intestinal cholesterol absorption is substantially reduced in mice deficient in both ABCA1 and ACAT2. J Lipid Res. (2005) 46:2423–31. 10.1194/jlr.M500232-JLR200 16150828

[80] Plösch T Kosters A Groen AK Kuipers F. The ABC of hepatic and intestinal cholesterol transport. Handb Exp Pharmacol. (2005) 170:465–82. 10.1007/3-540-27661-0_17 16596811

[81] Ye D Hoekstra M Out R Meurs I Kruijt JK Hildebrand RB. Hepatic cell-specific ATP-binding cassette (ABC) transporter profiling identifies putative novel candidates for lipid homeostasis in mice. Atherosclerosis. (2008) 196:650–8. 10.1016/j.atherosclerosis.2007.07.021 17727861

[82] Mulligan JD Flowers MT Tebon A Bitgood JJ Wellington C Hayden MR. ABCA1 is essential for efficient basolateral cholesterol efflux during the absorption of dietary cholesterol in chickens. J Biol Chem. (2003) 278:13356–66. 10.1074/jbc.M212377200 12551945

[83] Chiang JYL. Bile acid metabolism and signaling. Comp Physiol. (2013) 3:1191–212. 10.1002/cphy.c120023 23897684 PMC4422175

[84] Sakamoto K Kimura J. Mechanism of statin-induced rhabdomyolysis. J Pharmacol Sci. (2013) 123:289–94. 10.1254/jphs.13R06CP 24257439

[85] Bouitbir J Sanvee GM Panajatovic MV Singh F Krähenbühl S. Mechanisms of statin-associated skeletal muscle-associated symptoms. Pharmacol Res. (2020) 154:104201. 10.1016/j.phrs.2019.03.010 30877064

[86] Rabkin SW Lodha P Kong JY. Reduction of protein synthesis and statin-induced cardiomyocyte cell death. Cardiovasc Toxicol. (2007) 7:1–9. 10.1007/s12012-007-0003-7 17646677

[87] Cao P Hanai J Tanksale P Imamura S Sukhatme VP Lecker SH. Statin-induced muscle damage and atrogin-1 induction is the result of a geranylgeranylation defect. Faseb J. (2009) 23:2844–54. 10.1096/fj.08-128843 19406843 PMC2735363

[88] Camerino GM Tarantino N Canfora I De Bellis M Musumeci O Pierno S. Statin-induced myopathy: Translational studies from preclinical to clinical evidence. Int J Mol Sci. (2021) 22:2070. 10.3390/ijms22042070 33669797 PMC7921957

[89] Reidenberg MM. Statins, lack of energy and ubiquinone. Br J Clin Pharmacol. (2005) 59:606–7. 10.1111/j.1365-2125.2005.02359.x 15842563 PMC1884835

[90] Tuckow AP Jefferson SJ Kimball SR Jefferson LS. Simvastatin represses protein synthesis in the muscle-derived C_2_C1_2_ cell line with a concomitant reduction in eukaryotic initiation factor 2B expression. Am J Physiol Endocrinol Metab. (2011) 300:E564–70. 10.1152/ajpendo.00383.2010 21224482 PMC3064004

[91] Feng B Yao PM Li Y Devlin CM Zhang D Harding HP. The endoplasmic reticulum is the site of cholesterol-induced cytotoxicity in macrophages. Nat Cell Biol. (2003) 5:781–92. 10.1038/ncb1035 12907943

[92] Choy KW Murugan D Mustafa MR. Natural products targeting ER stress pathway for the treatment of cardiovascular diseases. Pharmacol Res. (2018) 132:119–29. 10.1016/j.phrs.2018.04.013 29684674

[93] Wang Y Xuan L Cui X Wang Y Chen S Wei C. Ibutilide treatment protects against ER stress induced apoptosis by regulating calumenin expression in tunicamycin treated cardiomyocytes. PLoS ONE. (2017) 12:e0173469. 10.1371/journal.pone.0173469 28399139 PMC5388464

[94] Lenna S Farina AG Martyanov V Christmann RB Wood TA Farber HW. Increased expression of endoplasmic reticulum stress and unfolded protein response genes in peripheral blood mononuclear cells from patients with limited cutaneous systemic sclerosis and pulmonary arterial hypertension. Arthritis Rheum. (2013) 65:1357–66. 10.1002/art.37891 23400395 PMC3636187

[95] Rao RV Peel A Logvinova A del Rio G Hermel E Yokota T. Coupling endoplasmic reticulum stress to the cell death program: role of the ER chaperone GRP78. FEBS Lett. (2002) 514:122–8. 10.1016/S0014-5793(02)02289-5 11943137 PMC3971841

[96] Andersson M Ellegård L Andersson H. Oat bran stimulates bile acid synthesis within 8 h as measured by 7α-hydroxy-4-cholesten-3-one. Am J Clin Nutr. (2002) 76:1111–6. 10.1093/ajcn/76.5.1111 12399287

[97] Goessling W Sadler KC. Zebrafish: an important tool for liver disease research. Gastroenterology. (2015) 149:1361–77. 10.1053/j.gastro.2015.08.034 26319012 PMC4762709

[98] Landgraf K Schuster S Meusel A Garten A Riemer T Schleinitz D. Short-term overfeeding of zebrafish with normal or high-fat diet as a model for the development of metabolically healthy versus unhealthy obesity. BMC Physiol. (2017) 17:4–4. 10.1186/s12899-017-0031-x 28327129 PMC5361797

[99] de Oliveira LB Leonardo AS de Lima EM. Macro- and microstructural descriptions of the zebrafish (Danio rerio) liver. Folia Morphol. (2016) 75:382–7. 10.5603/FM.a2016.0009 26916203

[100] Park KH Ye ZW Zhang J Kim SH. Palmitic acid-enriched diet induces hepatic steatosis and injury in adult zebrafish. Zebrafish. (2019) 16:497–504. 10.1089/zeb.2019.1758 31355732 PMC6916734

[101] Sun S Castro F Monroig Ó Cao X Gao J. fat-1 transgenic zebrafish are protected from abnormal lipid deposition induced by high-vegetable oil feeding. Appl Microbiol Biotechnol. (2020) 104:7355–65. 10.1007/s00253-020-10774-x 32676712

[102] Wolf JC Wolfe MJ. A brief overview of nonneoplastic hepatic toxicity in fish. Toxicol Pathol. (2005) 33:75–85. 10.1080/01926230590890187 15805058

[103] Watts SA Powell M D'Abramo LR. Fundamental approaches to the study of zebrafish nutrition. ILAR J. (2012) 53:144–60. 10.1093/ilar.53.2.144 23382346 PMC4064678

[104] Fowler LA Williams MB D'Abramo LR Watts SA. Chapter 33 - Zebrafish nutrition—moving forward. In: Cartner C Eisen JS Farmer SC Guillemin KJ Kent GML Sanders E, editors. The Zebrafish in Biomedical Research. S Academic Press (2020).

[105] Choi JS Kim H Jung MH Hong S Song J. Consumption of barley beta-glucan ameliorates fatty liver and insulin resistance in mice fed a high-fat diet. Mol Nutr Food Res. (2010) 54:1004–13. 10.1002/mnfr.200900127 20112296

[106] Tziomalos K. Lipid-lowering agents in the management of nonalcoholic fatty liver disease. World J Hepatol. (2014) 6:738–44. 10.4254/wjh.v6.i10.738 25349644 PMC4209418

[107] Dailey MJ. Nutrient-induced intestinal adaption and its effect in obesity. Physiol Behav. (2014) 136:74–8. 10.1016/j.physbeh.2014.03.026 24704111 PMC4182169

[108] Rong S McDonald JG Engelking LJ. Cholesterol auxotrophy and intolerance to ezetimibe in mice with SREBP-2 deficiency in the intestine. J Lipid Res. (2017) 58:1988–98. 10.1194/jlr.M077610 28630260 PMC5625122

[109] Lee H Yoo YS Lee D Song EJ. Cholesterol induces cardiac hypertrophy by activating the AKT pathway. J Steroid Biochem Mol Biol. (2013) 138:307–13. 10.1016/j.jsbmb.2013.07.008 23907017

[110] Wang B Rong X Palladino END Wang J Fogelman AM Martín MG. Phospholipid remodeling and cholesterol availability regulate intestinal stemness and tumorigenesis. Cell Stem Cell. (2018) 22:206–20. 10.1016/j.stem.2017.12.017 29395055 PMC5807072

[111] Le Roy T Lécuyer E Chassaing B Rhimi M Lhomme M Boudebbouze S. The intestinal microbiota regulates host cholesterol homeostasis. BMC Biol. (2019) 17:94. 10.1186/s12915-019-0715-8 31775890 PMC6882370

[112] Begley M Hill C Gahan CG. Bile salt hydrolase activity in probiotics. Appl Environ Microbiol. (2006) 72:1729–38. 10.1128/AEM.72.3.1729-1738.2006 16517616 PMC1393245

[113] Sridevi N Vishwe P Prabhune A. Hypocholesteremic effect of bile salt hydrolase from *Lactobacillus buchneri* ATCC 4005. Food Res Int. (2009) 42:516–20. 10.1016/j.foodres.2009.02.016

[114] Lefebvre P Cariou B Lien F Kuipers F Staels B. Role of bile acids and bile acid receptors in metabolic regulation. Physiol Rev. (2009) 89:147–91. 10.1152/physrev.00010.2008 19126757

[115] Wen J Mercado GP Volland A Doden HL Lickwar CR Crooks T. Fxr signaling and microbial metabolism of bile salts in the zebrafish intestine. Sci. Adv. (2021) 7: eabg1371. 10.1126/sciadv.abg1371 34301599 PMC8302129

[116] Gilissen L van der Meer IM Smulders MJM. Why oats are safe and healthy for celiac disease patients. Med Sci. (2016) 4:21. 10.3390/medsci4040021 29083384 PMC5635790

[117] Gangopadhyay N Hossain MB Rai DK Brunton NP. A review of extraction and analysis of bioactives in oat and barley and scope for use of novel food processing technologies. Molecules. (2015) 20:10884–909. 10.3390/molecules200610884 26076110 PMC6272431

[118] Harasym J Zyła E Dziendzikowska K Gromadzka-Ostrowska J. Proteinaceous residue removal from oat β-glucan extracts obtained by alkaline water extraction. Molecules. (2019) 24:1729. 10.3390/molecules24091729 31058866 PMC6539924

[119] Grundy MM Quint J Rieder A Ballance S Dreiss CA Butterworth PJ. Impact of hydrothermal and mechanical processing on dissolution kinetics and rheology of oat β-glucan. Carbohydr Polym. (2017) 166:387–97. 10.1016/j.carbpol.2017.02.077 28385246 PMC5388193

[120] Wang Y Ames NP Tun HM Tosh SM Jones PJ Khafipour E. High molecular weight barley β-glucan alters gut microbiota toward reduced cardiovascular disease risk. Front Micobiol. (2016) 7:129. 10.3389/fmicb.2016.00129 26904005 PMC4748052

