# Supplementary material for: Management of Hypercholesterolemia Through Dietary ß-glucans–Insights From a Zebrafish Model
Source: Front Nutr. 2022 Jan 12;8:797452. doi: 10.3389/fnut.2021.797452 (PMC8790573; doi:10.3389/fnut.2021.797452)
Supplement: Supplementary file 1 [file Data_Sheet_1.pdf]

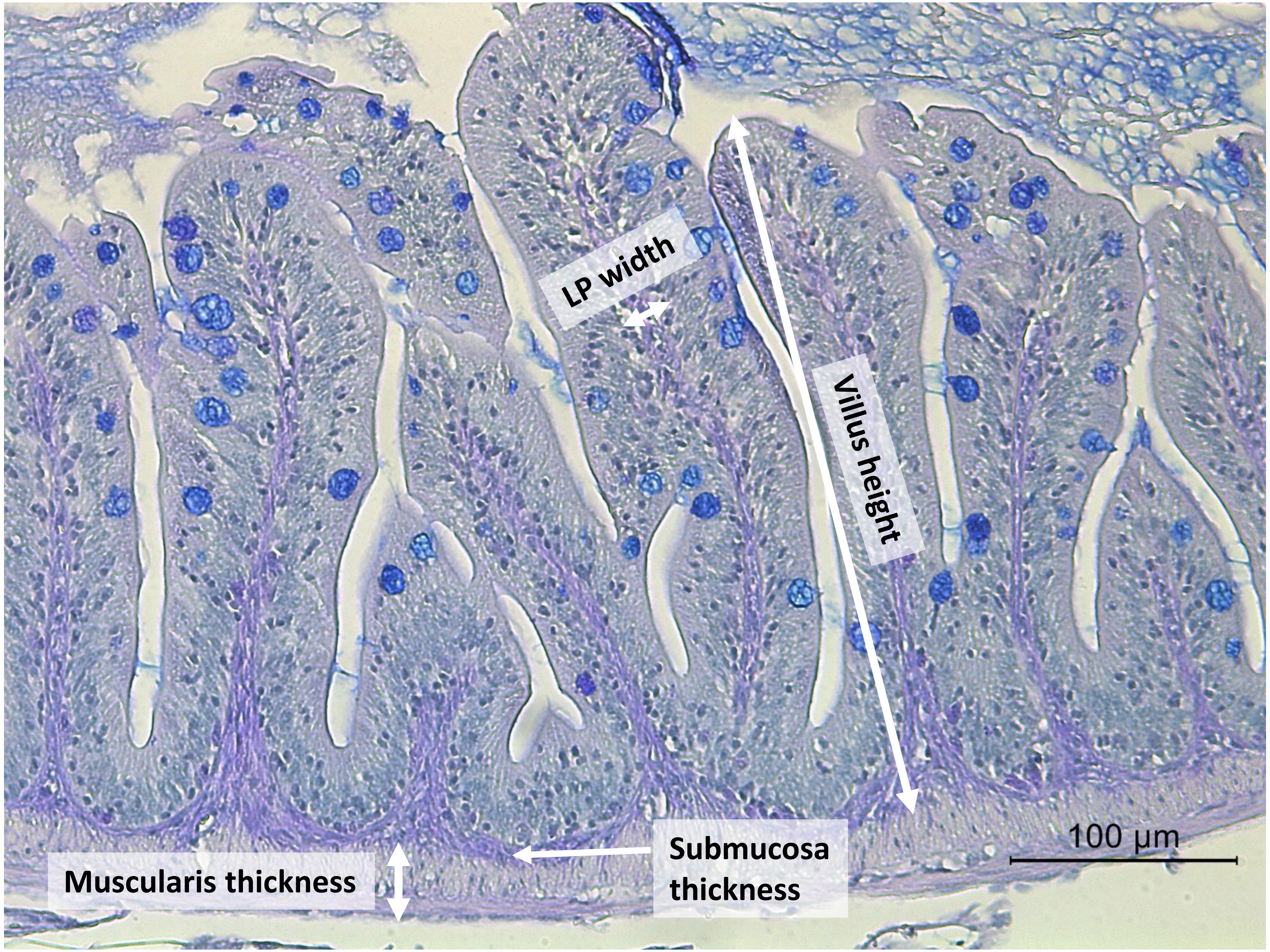

Supplementary figure 1: Illustration of the measurement strategy to assess the intestinal histological changes.

Scale bar = 100 µm.

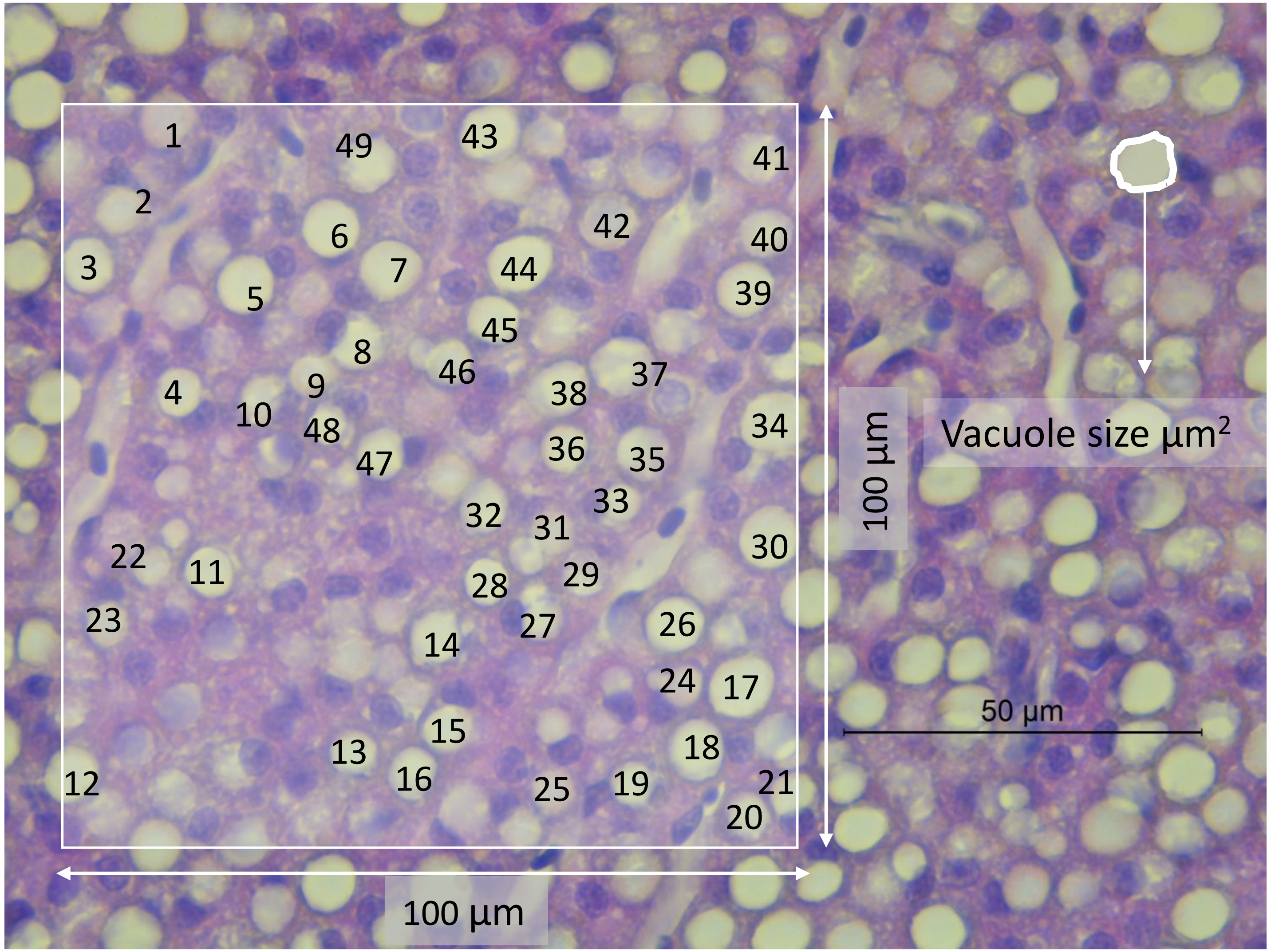

Supplementary Figure 2: Illustration of the histological measurement strategy to assess the vacuole area and number in the liver. Scale bar = 50 µm.

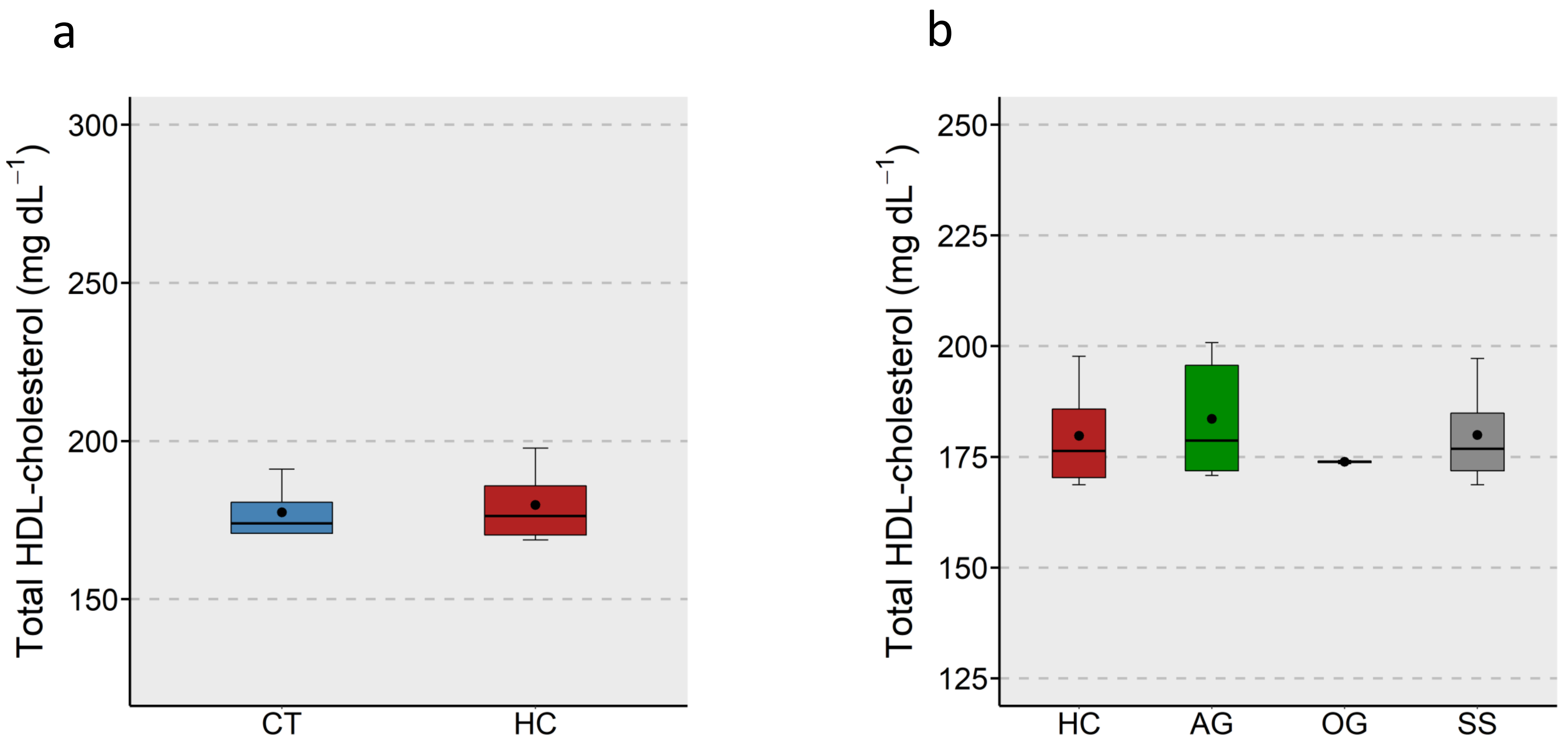

Supplementary Figure 3: Total HDL-cholesterol levels in the plasma of zebrafish. (a) Boxplots show HDL-cholesterol (mg dL^{-1}) in the plasma of zebrafish fed the control (CT) and high cholesterol (HC) diets for a period of 12 weeks. (b) Boxplots show HDL-cholesterol in the plasma of zebrafish fed HC diet, or HC diet further supplemented with algal glucan (AG), oat glucan (OG) and simvastatin (SS), for a period of 12 weeks. Black dots inside each box indicate the mean values of the corresponding groups.

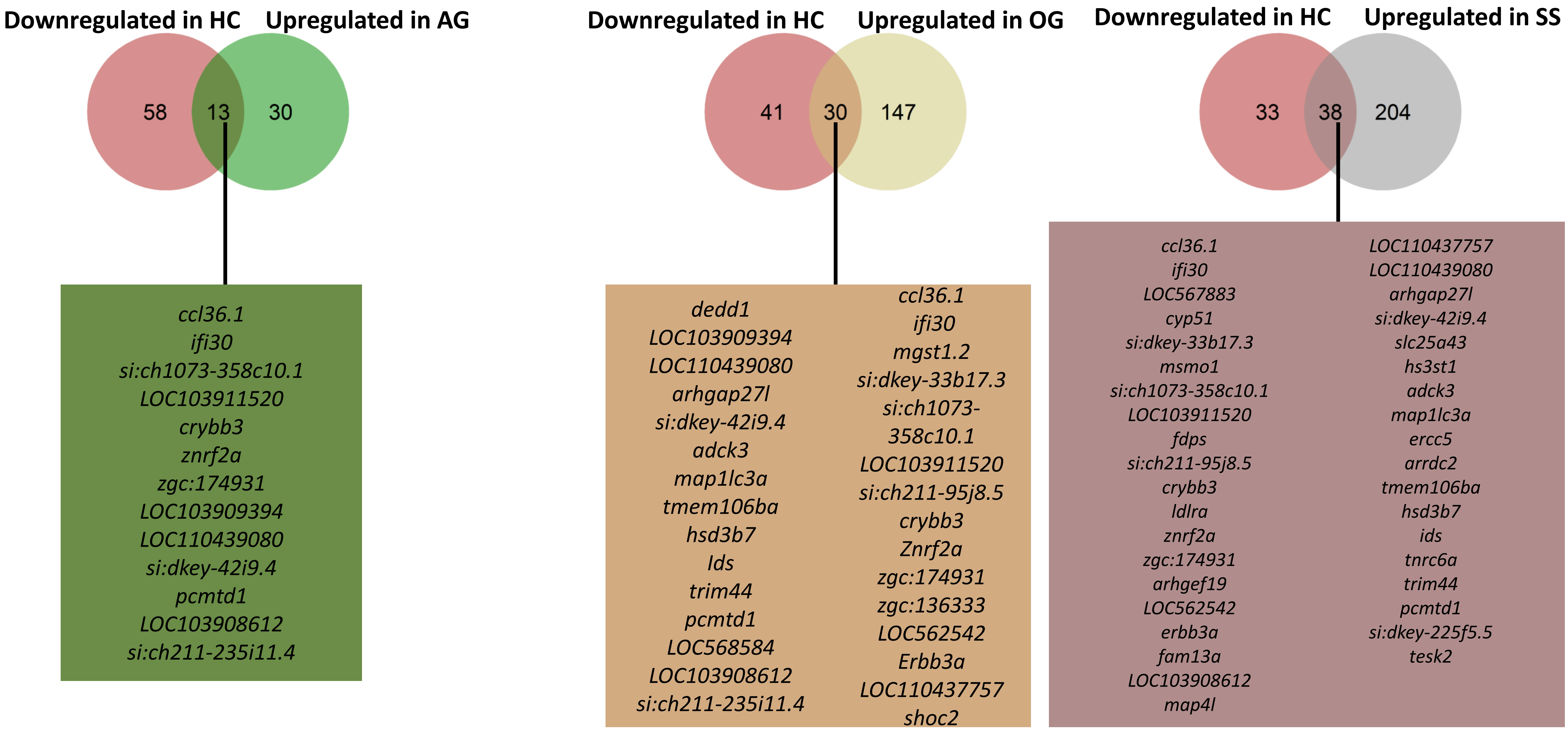

Supplementary Figure 4: Genes that were affected by the experimental diets. Pink ellipse includes the genes that were downregulated in the high cholesterol (HC) group compared to the control (CT) group. Green, yellow and grey colour ellipses include genes that were upregulated (compared to the HC group) in the AG, OG and SS groups, respectively. Thirteen, 30 and 38 genes that were downregulated in the HC group were upregulated in the respective groups.

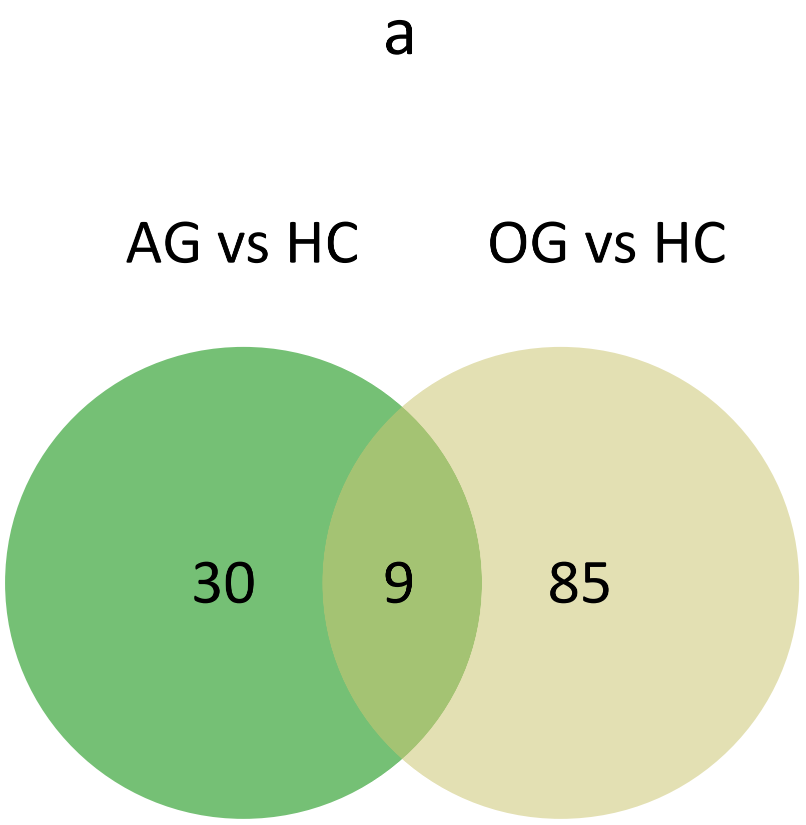

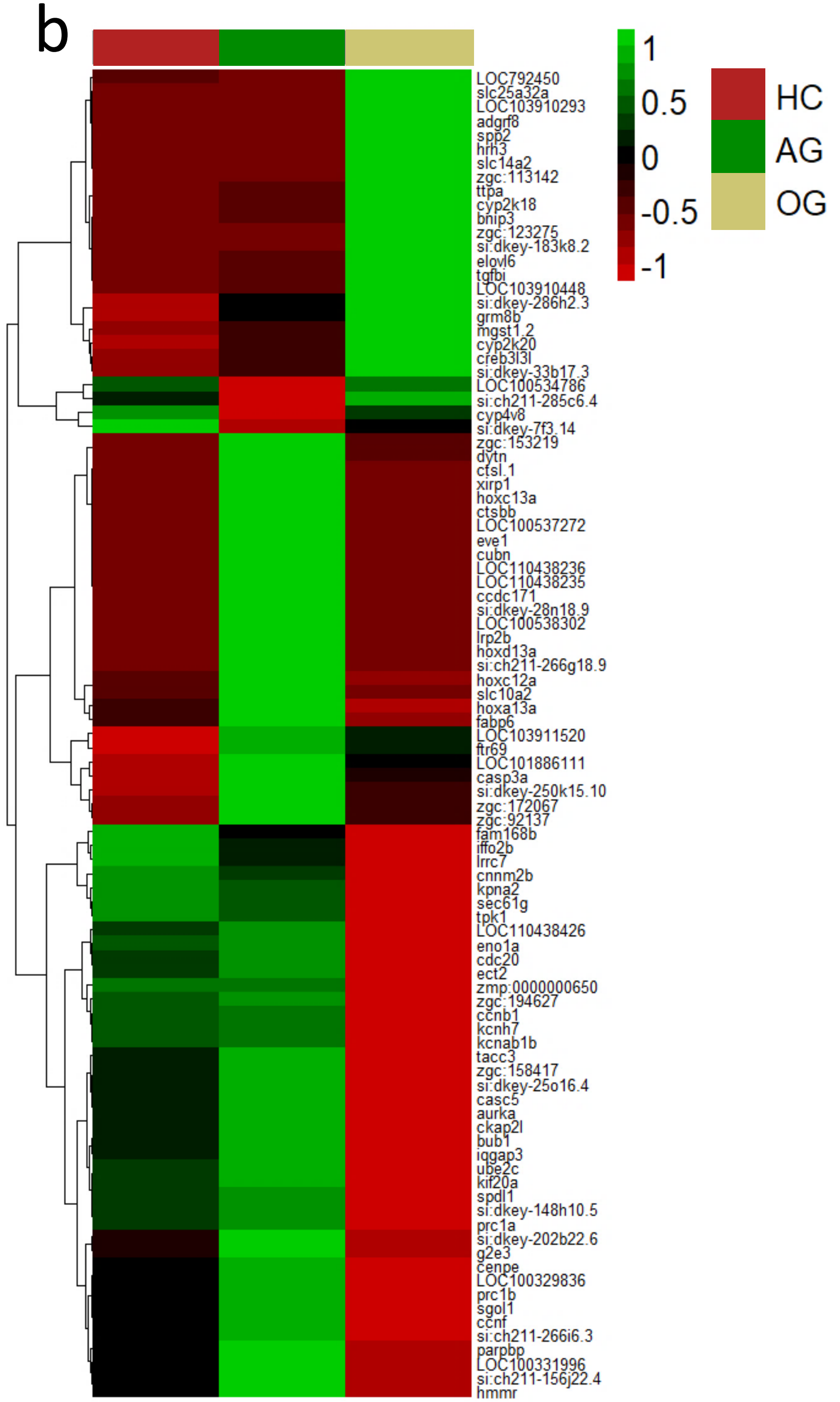

Supplementary Figure 5: (a) Unique differentially expressed genes (|Log_2 fold-change| ≥ 2.5, q-value < 0.05) in AG and OG groups, compared to the HC group. (b) Heatmap of group-wise average normalized counts of selected genes in HC, AG and OG (genes which showed a contrast in the normalized expression in AG and OG are shown).

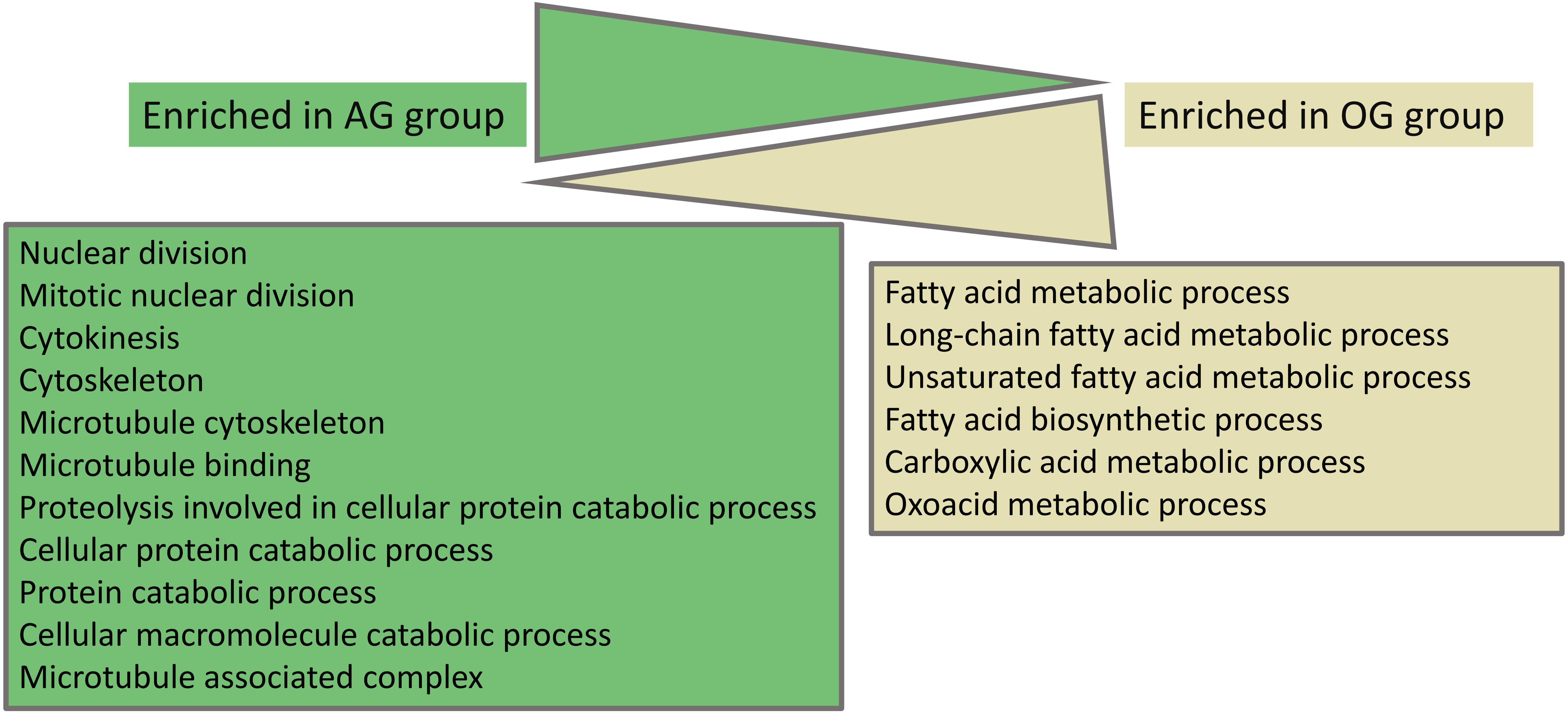

Supplementary Figure 6: Gene ontology analysis of unique differentially expressed genes ($|Log_2$ fold-change$| \geq 2.5$, q-value $<$ 0.05) obtained from AG vs HC and OG vs HC comparisons. Enriched terms in the boxes colored green and yellow belong to genes that had higher fold changes in the AG and OG groups, respectively.

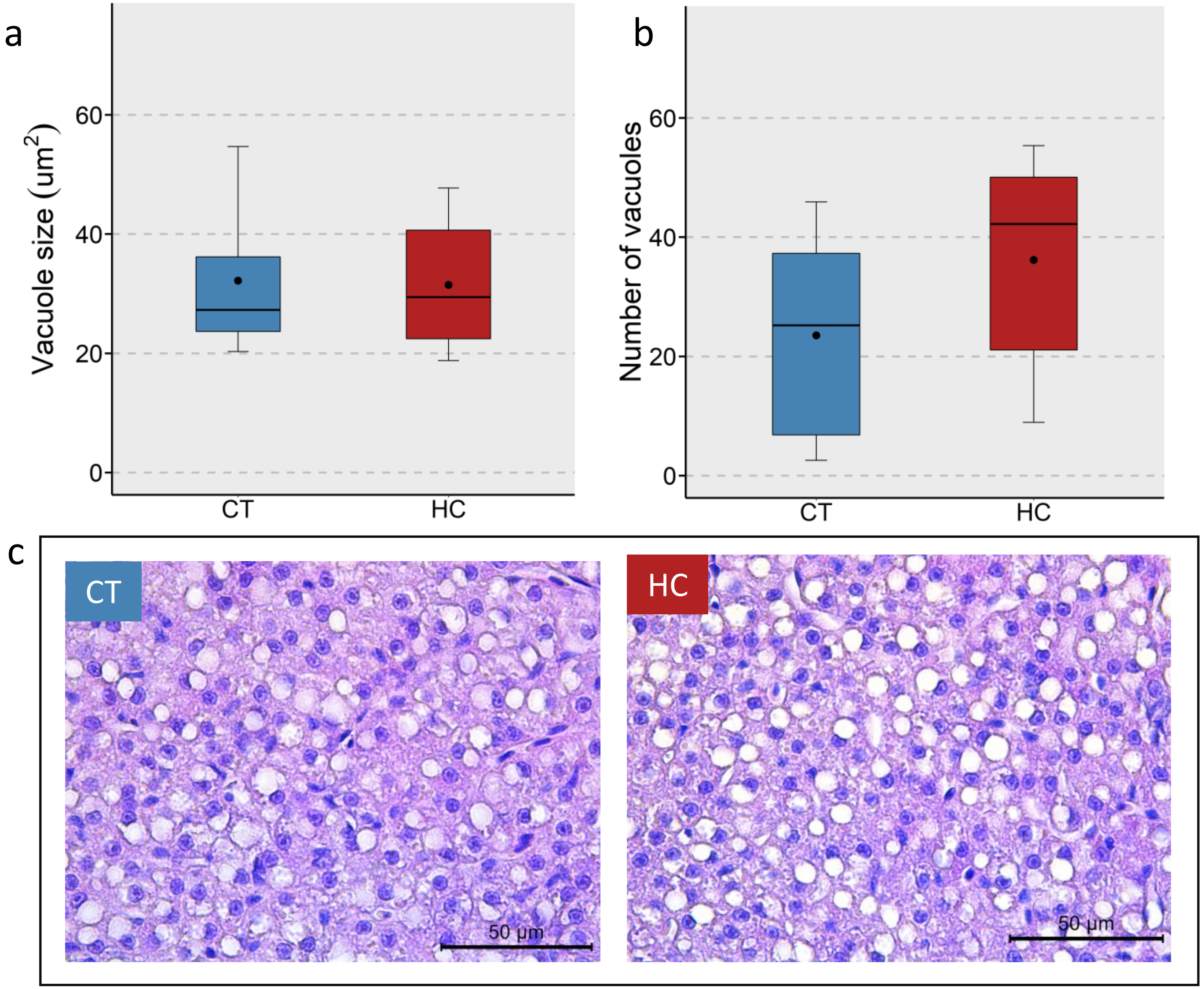

Supplementary Figure 7: Lipid accumulation in the liver of zebrafish fed control (CT) and high cholesterol (HC) diets. Boxplots show average size (a) and number (b) of vacuoles in the liver of zebrafish fed the experimental diets for a period of 12 weeks. Black dots inside each box indicate the mean values of the corresponding groups. (c) Representative images of the liver zebrafish fed CT or HC diet. Each treatment group consisted of eight biological replicates. Scale bar = 50 µm.

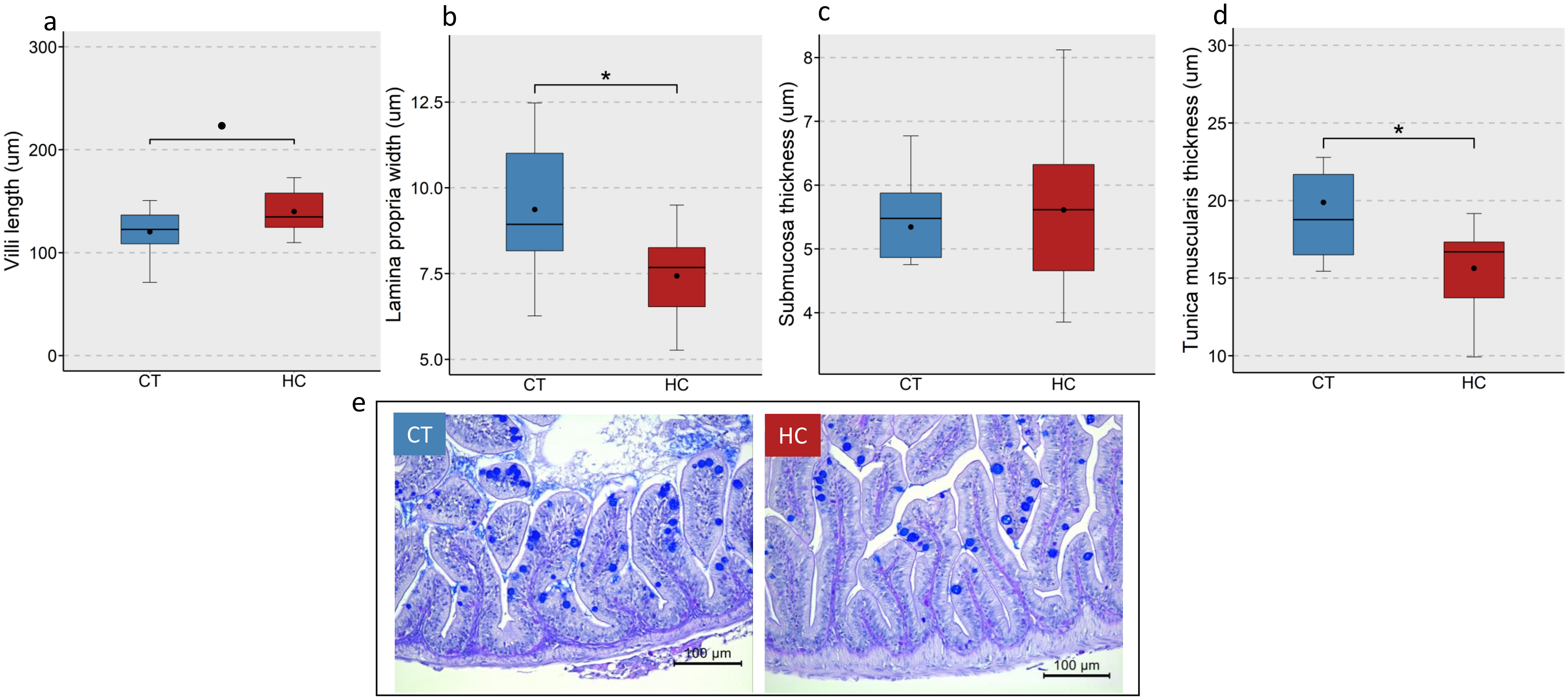

Supplementary Figure 8: Micromorphology and feature indices of the intestine of zebrafish. Boxplots show villi length (a), lamina propria width (b), submucosa thickness (c) and tunica muscularis thickness (d) in the mid intestine of zebrafish fed control (CT) or high cholesterol (HC) diets for a period of 12 weeks. Black dots inside each box indicate the mean values of the corresponding groups. (e) Representative images of the mid intestine of zebrafish fed CT or HC diet. * indicates $p < 0.05$, and • indicates $p < 0.1$. Each treatment group consisted of eight biological replicates. Scale bar = 100 µm

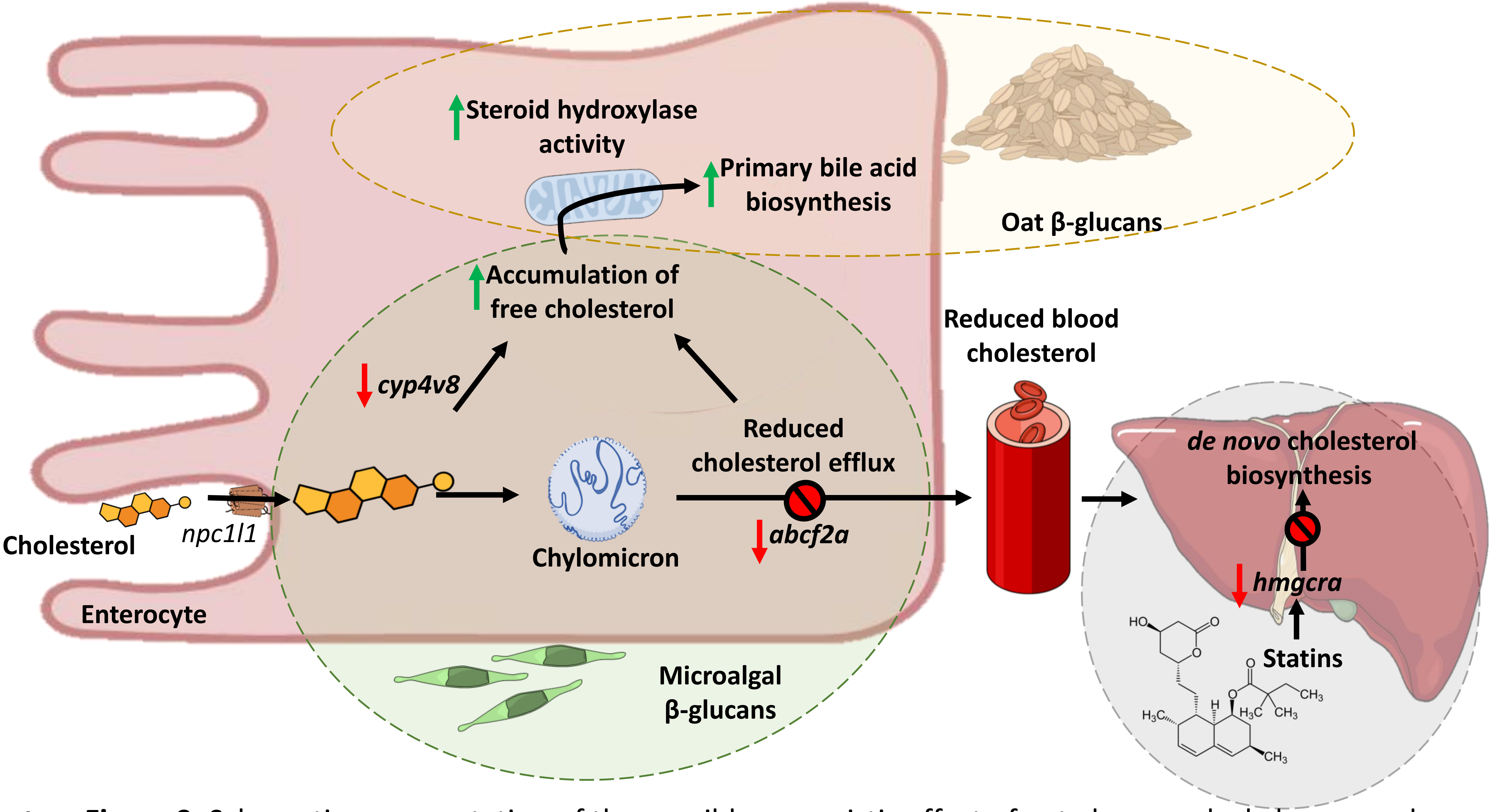

Supplementary Figure 9: Schematic representation of the possible synergistic effect of oat glucans, algal glucans and simvastatin. The algal glucan likely increased the accumulation of free cholesterol in the enterocytes whereas the oat glucan likely promoted bile acid biosynthesis. Yellow, green and grey ellipses indicate the effect of oat β-glucan, algal β-glucan and simvastatin, respectively.
